# Integrating Dynamical Systems Modeling with Spatiotemporal scRNA-Seq Data Analysis

**DOI:** 10.3390/e27050453

**Published:** 2025-04-22

**Authors:** Zhenyi Zhang, Yuhao Sun, Qiangwei Peng, Tiejun Li, Peijie Zhou

**Affiliations:** 1 School of Mathematical Sciences, Peking University, Beijing 100871, China; zhenyizhang@stu.pku.edu.cn (Z.Z.); qiangwei_peng@stu.pku.edu.cn (Q.P.); 2 Center for Machine Learning Research, Peking University, Beijing 100871, China; lh13210817312@gmail.com; 3 Laboratory of Mathematics and Its Applications (LMAM), Peking University, Beijing 100871, China; 4 Center for Quantitative Biology, Peking University, Beijing 100871, China; 5 National Engineering Laboratory for Big Data Analysis and Applications, Peking University, Beijing 100871, China; 6 AI for Science Institute, Beijing 100080, China

**Keywords:** single-cell RNA sequencing, spatiotemporal dynamics, computational modeling, cellular trajectories

## Abstract

Understanding the dynamic nature of biological systems is fundamental to deciphering cellular behavior, developmental processes, and disease progression. Single-cell RNA sequencing (scRNA-seq) has provided static snapshots of gene expression, offering valuable insights into cellular states at a single time point. Recent advancements in temporally resolved scRNA-seq, spatial transcriptomics (ST), and time-series spatial transcriptomics (temporal-ST) have further revolutionized our ability to study the spatiotemporal dynamics of individual cells. These technologies, when combined with computational frameworks such as Markov chains, stochastic differential equations (SDEs), and generative models like optimal transport and Schrödinger bridges, enable the reconstruction of dynamic cellular trajectories and cell fate decisions. This review discusses how these dynamical system approaches offer new opportunities to model and infer cellular dynamics from a systematic perspective.

## 1. Introduction

Understanding the dynamic change of biological systems has played a central role in life sciences, with important applications in developmental biology, disease modeling, and medicine [1,2,3,4,5]. One key framework for understanding these dynamic processes is Waddington’s developmental landscape [6,7,8], which illustrates how cells navigate various potential fates as they differentiate during development. However, how to construct such developmental landscapes or understand the cellular dynamics within the biological systems, presents a significant challenge. To fully understand these complex cellular transitions, a deep understanding of gene expression at the single-cell level is essential. Advancements in high-throughput sequencing technologies have enabled unprecedented resolutions into the molecular signatures of individual cells, with single-cell RNA sequencing (scRNA-seq) emerging as a revolutionary tool [9,10,11]. scRNA-seq allows for the dissection of cellular heterogeneity and the identification of transcriptional programs underlying complex biological processes, offering a snapshot of gene expression in single cells at a given moment. Despite its powerful capabilities, traditional scRNA-seq provides only a static picture of gene expression across individual cells, missing the temporal information for understanding how cells transition through different states.

In recent years, the development of temporally resolved scRNA-seq technologies has begun to gain increasing attention, enabling the capture of gene expression profiles across multiple time points [4,12,13]. Another breakthrough in transcriptomics is spatial transcriptomics (ST), which integrates spatial context into gene expression data by mapping RNA profiles within tissue architectures [14,15,16,17,18,19,20,21]. When combined with temporal resolution, this approach leads to temporally resolved spatial transcriptomics (temporal-ST), which provides an enhanced tool for studying the spatiotemporal dynamics of single cells [22].

Extracting meaningful dynamical features from spatiotemporal single-cell transcriptomic data remains a significant challenge. Since the inherently destructive nature of single-cell sequencing, each cell can only be measured once during the dynamical process. As a result, continuous dynamics cannot be directly obtained from the data. Even with temporally resolved single-cell RNA sequencing, we can only obtain unpaired gene expression snapshots at discrete time points, capturing cell distribution changes over time rather than the continuous movement of individual cells. Consequently, inferring cell-state transitions and dynamic regulatory mechanisms from such snapshot-based data necessitates computational modeling approaches, which is an important problem in computational system biology and has gained increasing importance.

To address the challenges, numerous computational frameworks have been developed. For single-cell transcriptomics data, several methods have been proposed to approximate cellular trajectories and dissect dynamic cellular states. Pseudotime inference methods [23,24,25], for instance, arrange snapshot data along an inferred developmental axis, offering a continuous perspective of cell-state transitions over time. In addition, RNA velocity analysis [26,27,28,29,30] has emerged as a powerful tool for understanding cellular dynamics by leveraging splicing kinetics to infer the direction of future gene expression changes. Recently, with the development of temporally resolved sequencing technology, there has been a growing interest in dissecting single-cell dynamics from multiple snapshot data. Simultaneously, the development of generative modeling techniques, such as diffusion models [31,32,33,34], optimal transport theory [13,35,36], flow-based model [37,38], and the Schrödinger bridge problem [39,40] have emerged as key mathematical frameworks for modeling distribution transitions in dynamic biological systems. More recently, the rapid development of spatial transcriptomics has opened exciting new avenues for integrating spatial and temporal data. Extending these computational methods to the ST data and capturing spatiotemporal cellular transitions also has inspired many recent kinds of research [41].

Recently, many reviews have provided comprehensive summaries of the methods and advancements in the study of single-cell dynamics. For example, ref. [42] reviewed various pseudotime inference methods. Ref. [43] conducted a comprehensive benchmarking study on pseudotime inference methods. Refs. [27,42,44] reviewed RNA velocity methods in single-cell transcriptomics. [27] also discussed the limitations and potential extensions of RNA velocity. Refs. [4,5,13,45] provided an in-depth analysis of the application of optimal transport theory in single-cell or spatial omics data. Additionally, refs. [1,3] examined various perspectives on cellular dynamics, exploring how the reconstruction of cell states and energy landscapes can contribute to our understanding of cellular behavior and development. The current review takes a distinct perspective by systematically discussing modeling strategies for different types of data from a dynamical modeling perspective, aiming to unify and expand upon the current methodologies in the field.

This paper mainly focuses on how dynamic insights can be extracted from high-resolution biological data, including scRNA-seq, temporally resolved scRNA-seq, spatial transcriptomics (ST), and temporally resolved spatial transcriptomics. We examine how key concepts from dynamical systems modeling—such as Markov chains, stochastic differential equations (SDEs), ordinary differential equations (ODEs), and partial differential equations (PDEs)—can be effectively applied to the analysis of cellular processes reflected in these high-dimensional data. Furthermore, we explore the application of emerging generative modeling techniques, including optimal transport theory, flow matching, and the Schrödinger bridge problem, as approaches for inferring spatiotemporal cellular trajectories and transitions. By focusing on these modeling strategies, this review aims to provide a systematic framework for understanding cellular dynamics across different types of data, thus advancing the study of spatiotemporal biological processes.

This paper is organized as follows: In Section 2, we provide an overview of the data and models, laying the foundation for understanding the types of biological data and the mathematical frameworks. Section 3 delves into the dynamic modeling of single-cell transcriptomics, with a focus on both single-cell RNA sequencing (scRNA-seq) and temporal-scRNA-seq. In Section 4, we explore the dynamic modeling of spatial transcriptomics, examining both snapshot-based and temporally resolved approaches to analyze the spatial and temporal dynamics of gene expression. Section 5 discusses the extensions, challenges, and future directions in the field, highlighting the key limitations and opportunities for advancing the study of cellular dynamics. Finally, we summarize the insights and outline potential areas for future research in Section 6.

## 2. Overview of the Data and Models

**Figure 1:**
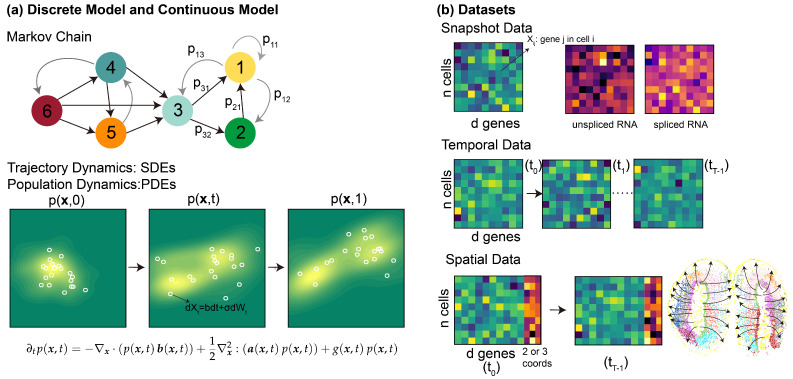
**Overview of the data and models.** (**a**) Discrete and Continuous Model: The discrete model constructs Markov chains between cells with dynamics encoded in a transition matrix, while the continuous model describes single-cell motion via stochastic differential equations (SDEs) and cell population dynamics through a corresponding partial differential equation (PDE). (**b**) Datasets: Snapshot data is an n×g matrix X (*n*: cell count, *g*: gene count); temporal data provides gene expression matrices $X_{i}$ at time points i∈{0,…,T−1}; spatial data additionally records coordinates for each cell in $X_{i}$.

In this section, we provide preliminary background on the structure of the scRNA-seq data as well as the mathematical models to describe dynamic cellular processes. An overview of the data and models is provided in Figure 1.

### 2.1. Spatiotemporal scRNA-Seq Data

Single-cell RNA sequencing (scRNA-seq) has emerged as a prevalent tool for dissecting cellular heterogeneity by providing high-resolution snapshots of gene expression profiles at the individual cell level. Traditionally, scRNA-seq experiments capture only single-time-point data, that is, a static “snapshot” of the cellular landscape. Recent advances in technologies have enhanced the spatiotemporal resolutions of the datasets, enabling finer resolutions to investigate the underlying dynamic biological processes such as development, differentiation, and disease progression. Below we describe the various types of scRNA-seq datasets as inputs to infer spatiotemporal dynamics through the dynamical systems models.

#### 2.1.1. Snapshot scRNA-Seq Data

In snapshot RNA sequencing (RNA-seq) data, gene expression is measured across multiple cells at a single time point. The gene expression matrix is represented as $X\in R^{n\times d}$, where X denotes the count matrix of gene expression, *n* is the number of cells or spots, and *d* is the number of genes measured. Each entry $X_{ij}$ in X represents the expression level of gene *j* in cell *i*, typically measured as the number of mRNA molecules (transcripts) for that gene in the corresponding cell. Additionally, the total RNA-seq data can further be separated into counts for spliced and unspliced transcripts, useful in certain analyses such as the RNA velocity model described below. The spliced and unspliced counts are represented as $U\in R^{n\times d}$ and $S\in R^{n\times d}$, respectively, denoting the matrices of unspliced and spliced counts for the *n* cells or spots. Over time, unspliced RNA (u) can undergo splicing process to become spliced RNA (s).

#### 2.1.2. Temporally and Spatially Resolved scRNA-Seq

Recently, a growing number of temporally resolved scRNA-seq datasets have been generated, where single-cell measurements are performed at multiple time points during a dynamic process. Such datasets could offer deeper insights into how cell populations evolve over time [4,12,13].

For temporally resolved scRNA-seq dataset, at each fixed time point i∈{0,⋯,T−1}, the gene expression matrix is represented as $X_{i}\in R^{n_{i}\times d}$, where $X_{i}$ denotes the matrix of gene expression data, $n_{i}$ is the number of cells at time *i*, and *d* is the number of genes. Notably, the gene expression data across time points are unpaired and can be assumed to be sampled from a distribution from a certain time point.

The development of spatial transcriptomics (ST) technology allows gene expression to be captured alongside spatial coordinates [14,15,16,17,18,19,20,21]. ST methods are broadly divided into image-based and sequencing-based approaches. Image-based techniques [19,20,21] detect hundreds to thousands of genes with cellular or sub-cellular resolution, while sequencing-based methods [15,16,17,18] allow whole-transcriptome analysis but are usually limited to spot-level resolution. Advances like Stereo-seq [17] and 10x Visium HD [18] have significantly improved spatial resolution to single-cell or even subcellular precision.

Similarly to temporally resolved scRNA-seq data, ST time series data could be represented as $\left(Z_{\left(0:K\right)},X_{\left(0:K\right)}\right)$ at $t_{0},t_{1}\ldots t_{K}$ totaling *K* time points, and the number of cells in each observation is $n_{0},n_{1}\ldots n_{K}$. In addition to the gene expression matrices $X_{i}\in R^{n_{i}\times d}$, the associated spatial coordinate matrices $Z_{i}\in R^{n_{i}\times 2}$ or $R^{n_{i}\times 3}$ represent the spatial coordinates (2D or 3D) of each sequenced cell (or spot), respectively.

### 2.2. Models for Cell-State Transitions

In computational systems biology, several modeling strategies have been formulated to quantify the cell-state transition dynamics. In general, they could be categorized into two types: discrete models, which are usually defined on observed samples and evolve in discrete time steps, as well as continuous models, which are extrapolated into the continuous cell state space and described by differential equation models.

#### 2.2.1. Discrete Dynamics: Markov Chain Model

**Random walk** or **Markov chain** models are simple yet powerful tools for studying stochastic dynamic processes, particularly in the context of complex systems such as gene expression dynamics and cell trajectories. In these models, a system evolves over time as a series of transitions between discrete states, where each state corresponds to a possible configuration or position in the system (such as a specific gene expression profile or a cell’s position in a developmental trajectory). The transitions between states are governed by transition probabilities, which can be represented in the form of a transition matrix P. The transition matrix is defined as $P_{ij}=\frac{W_{ij}}{W_{i}},$ where $P_{ij}$ represents the probability of transitioning from cell state *i* to state *j*, and $W_{ij}$ is the weight (or similarity) between cells *i* and *j* in a weighted graph, and $W_{i}=\sum_{k}W_{ik}$ is the degree of cell *i*. The weights $W_{ij}$ typically reflect some measure of similarity or distance between the corresponding gene expression profiles of the cells, or induced from other quantities such as RNA velocity or optimal transport plan.

The **stationary distribution** $\pi =\left(\pi_{1},\pi_{2},\cdots ,\pi_{n}\right)$ of the Markov chain is a probability distribution over the states that remains unchanged under the dynamics of the chain. In other words, the distribution is invariant under the transition probabilities, and we have $\pi_{i}=\sum_{j}P_{ij}\pi_{j}.$ If the cellular state graph is undirected (for example, induced by gene expression similarity), i.e., W is a symmetric matrix and we have the expression $\pi_{i}=\frac{W_{i}}{\sum_{i}W_{i}}$, then the stationary Markov chain is in detailed balance such that $P_{ij}\pi_{i}=P_{ji}\pi_{j}$.

A more realistic assumption in biology is that the state-transition graph can be directional (for example, induced by RNA velocity or optimal transport discussed below), with the cells ultimately reaching terminal states such as fully differentiated or mature cell types. In this setup, **recurrent states** represent the final, stable cell types or fates that the system eventually reaches. Once a cell enters one of these recurrent states, it remains there, similar to how a fully differentiated cell does not revert back to an undifferentiated or less specialized state. On the other hand, **transient states** correspond to intermediate stages of cellular development, such as precursor or progenitor cells that are still undergoing differentiation or division. These cells are in transition, with the potential to eventually reach one of the recurrent, stable cell types. The transition matrix governing this system can be partitioned into blocks that reflect these different types of cell states. Specifically, the matrix P can be written as the canonical form

(1) $$P=\left[\begin{array}{cc} \tilde{P} & 0\\ S & Q \end{array}\right].$$

Here, $\tilde{P}$ corresponds to the transitions between recurrent (terminal) states, where once a cell reaches these states, it remains there in absorbing states. Q represents transitions between transient states (cells that are still in intermediate stages of differentiation or cell cycle). S denotes the transitions from transient to recurrent states, representing cells’ eventual differentiation or maturation into their terminal stable types. Since recurrent states are absorbing, the upper right block of the matrix is zero, indicating no transitions from recurrent to transient states.

#### 2.2.2. Continuous Dynamics: From Trajectories to Population Dynamics

In modeling cellular dynamics, we are interested in both the trajectories of individual cells and the distribution of cell states across a population. To capture the behavior of cells in response to both deterministic and stochastic influences, we can approach the problem from two perspectives: (1) **cellular trajectories**, which describe the path of individual cells over time, and (2) **population distribution**, which describes how the overall distribution of cell states evolves. The first perspective, trajectory-based models, provides insight into the detailed behavior of a single cell, often described through ordinary or stochastic differential equations (ODEs or SDEs). The second perspective, population-level models, focuses on the evolution of the density of cells across different states, typically captured by partial differential equations (PDEs). Together, these models offer a comprehensive understanding of how individual cell behaviors aggregate to produce population-level dynamics.

**Trajectory Dynamics: Stochastic Differential Equations (SDEs)** To model the evolution of individual cell trajectories, we consider that cellular dynamics can be governed by a stochastic differential equation (SDE). This accounts for both deterministic factors, such as gene expression regulation, and stochastic factors, like noise from cellular environments or molecular fluctuations. The SDE for the state $x_{t}$ of a single cell at time *t* is given by

(2) $$dx_{t}=b\left(x_{t},t\right)\;dt+\sigma \left(x_{t},t\right)\;dw_{t},$$

where $x_{t}\in R^{d}$ represents the state of the cell (e.g., gene expression profile) at time *t*, and $w_{t}\in R^{d}$ is the standard *d*-dimensional Brownian motion. The term b(x,t) represents the drift vector, which defines the deterministic flow of the system, while $\sigma \left(x,t\right)\in R^{d\times d}$ represents the diffusion coefficient matrix, which describes the random fluctuations in the system.

Specifically, when the diffusion coefficient σ(x,t) is zero, the system reduces to an ordinary differential equation (ODE), which describes the deterministic evolution of the cell state without random fluctuations. In this case, the evolution of the cell is entirely governed by the drift term b(x,t), and the system follows a deterministic trajectory. A useful concept in understanding the long-term behavior of the system is that of an *attractor*. In the context of the ODE, an attractor corresponds to a stable fixed point of the system, where the rate of change b(x,t) of the cell state x becomes zero. In cellular dynamics, such attractors can represent stable gene expression profiles, such as differentiated or quiescent states, where the cell remains in a stable state over time.

Furthermore, since b(x,t) is time-dependent, the system may exhibit *bifurcations*, where the qualitative characteristics of attractors could change with respect to time *t*. In cellular contexts, bifurcations are important for understanding processes like cell fate decisions, where a small change in the environment or internal signaling can push the cell toward a new distinct state (e.g., differentiation into a different cell type).

**Population Dynamics: Partial Differential Equations (PDEs)** To capture the evolution of the entire population of cells, we consider the density of cells with respect to their state x, represented by the probability density function p(x,t). The population distribution evolves according to a partial differential equation (PDE), which incorporates both deterministic and stochastic dynamics at the population level. As a simple model, the evolution of p(x,t) can be described by

(3) $$\partial_{t}p\left(x,t\right)=-\nabla_{x}\cdot \left(p(x,t)\;b(x,t)\right)+\frac{1}{2}\nabla_{x}^{2}:\left(a(x,t)\;p(x,t)\right)+g\left(x,t\right)\;p\left(x,t\right),$$

where $\nabla_{x}^{2}:\left(a(x,t)\;p(x,t)\right)=\sum_{ij}\partial_{ij}\left(a_{ij}\left(x,t\right)p\left(x,t\right)\right)$, and $a\left(x,t\right)=\sigma \left(x,t\right)\sigma^{T}\left(x,t\right)$ represents the diffusion matrix at the population level.

The terms on the right-hand side of the equation represent the key dynamics driving the population evolution. The *drift term* $\nabla_{x}\cdot \left(p(x,t)\;b(x,t)\right)$ quantifies the deterministic flow of the population, describing how cells move through different states based on the drift vector b(x,t). The *diffusion term*$\frac{1}{2}\nabla_{x}^{2}:\left(a(x,t)\;p(x,t)\right)$ models the spread of the population due to random fluctuations, where $a\left(x,t\right)=\sigma \left(x,t\right)\sigma^{T}\left(x,t\right)$ represents the diffusion matrix, capturing the effects of stochasticity. Finally, the *growth term* g(x,t)p(x,t) governs the birth and death rates of cells, modeling cell proliferation and mortality, thus controlling the population’s size and dynamics over time. When g(x,t)=0, the PDE reduces to the Fokker–Planck equation associated with the SDE in the Itô integral sense, describing the evolution of the probability density p(x,t) for the stochastic process defined by the SDE.

## 3. Dynamic Modeling of Single-Cell Transcriptomics

In this section, we describe the dynamical systems models for scRNA-seq datasets. We begin with methods for snapshot data, i.e., cells sequenced at a single time point, including pseudotime methods, discrete Markov chain methods, and continuous RNA velocity methods and their extensions. Next, we review methods targeted for temporally resolved scRNA-seq data, majorly based on various formulations and extensions of optimal transport (OT) based methods.

### 3.1. Snapshot Single-Cell RNA-Seq

A key challenge in using snapshot data to infer dynamic cellular trajectories lies in the inability to directly observe the temporal evolution of cells. The destructive nature of the measurement process, where cells are disassociated after sequencing, means that we lack direct access to the temporal trajectory of individual cells.

**Figure 2:**
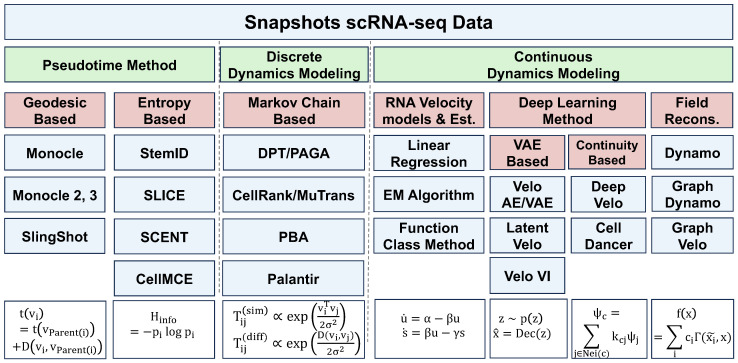
**Dynamic modeling of snapshot single-cell transcriptomics**.

When analyzing such a snapshot of “cell state ensembles”, several approaches have been developed to uncover the underlying dynamical processes. One popular type of method, **pseudotime**, ranks individual cells temporally based on the structure of the data manifold or prior biological knowledge. Other techniques focus on modeling stochastic dynamics over the point clouds of observed cells, yielding **discrete random walk analyses**. Additionally, **continuous differential equation models** have been proposed to infer the data-generating process of snapshot scRNA-seq dataset, to use these models for future dynamical predictions. In the following sections, we will explore these methods in more detail. An overview of these approaches is provided in Figure 2.

#### 3.1.1. Pseudotime Methods

Given snapshot data from single-cell sequencing, where the data matrix $X\in R^{n\times d}$, pseudotime assigns a positive real number for each cell to reflect its order during a dynamical process. Let $x_{i}\in R^{d}$ represent the state vector (such as mRNA expression) for the *i*-th cell. The pseudotime $t_{i}\in R$ is then a mapping from the state vector $x_{i}$ to a real number, i.e., $x_{i}\mapsto t_{i}$.

Pseudotime can be viewed from two perspectives. First, the **geodesic** perspective considers that the cell’s state is constrained by a limited number of biological pathways, limiting the evolution of the state to a low-dimensional manifold embedded in the high-dimensional gene expression space. Given an initial state $x_{0}$, the task of finding the mapping $x_{i}\mapsto t_{i}$ becomes equivalent to determining the length of the evolutionary path between $x_{0}$ and $x_{i}$ along this manifold. Several tools, such as Monocle, Slingshot, DPT, and PAGA, have been developed based on this idea. Second, the **entropy** perspective recognizes that during natural biological development, as cells differentiate, they become more specialized and lose their potential for further differentiation.

**Geodesic-Based Pseudotime** Geodesic -based pseudotime aims to reconstruct these trajectories by leveraging graph-based methods and principal curve algorithms [46]. Two widely used approaches include **Monocle** [23,24] and **Slingshot** [25], along with numerous other methods. Monocle focuses on ordering cells using a minimum spanning tree and a refined PQ tree approach, and Slingshot can handle multiple lineages and smooth pseudotime across branching events. Both methods offer insights into cellular dynamics, helping to uncover the paths cells take through different states.

Monocle estimates pseudotime through two main steps: (1) ordering cells and (2) assigning pseudotime values. First, each cell’s state is reduced to a *d*-dimensional vector $x_{i}\in R^{d}$ using Independent Component Analysis (ICA). A complete graph is created where vertices represent cells, and edges are weighted by Euclidean distance. Cell ordering relies on the minimum spanning tree (MST) of this graph, which is refined using a PQ tree to mitigate noise from sequencing. The tree is constructed by first identifying the longest path (diameter path), classifying vertices as decisive or indecisive, and recursively building the tree by ordering decisive vertices and handling indecisive vertices through new P nodes. Once ordered, pseudotime is calculated as

$$t\left(x_{i}\right)=t\left(x_{\mathrm{Parent}(i)}\right)+∥x_{i}-x_{\mathrm{Parent}(i)}∥,$$

where Parent(i) is the parent node of cell *i*, and the root node (selected based on prior knowledge) is initialized with pseudotime 0.

Slingshot estimates pseudotime across multiple lineages. It begins by clustering cells into *K* clusters and identifying lineages using the MST. After constructing the MST, a new lineage is formed at each branching point. Pseudotime is assigned using the principal curve algorithm, which involves projecting cells onto the curve, computing arc lengths, and smoothing iteratively. To handle inconsistent pseudotime across multiple lineages, Slingshot modifies the standard principal curve approach. It initializes the curve for each lineage through the centroids of its clusters, assigns weights to cells in multiple lineages based on projection distances, and constructs an average curve for smooth transitions at shared cell regions. The average curve is defined as

$$c_{\mathrm{avg}}\left(t\right)=\frac{1}{M}\sum_{m=1}^{M}c_{m}\left(t\right),$$

where $c_{m}$ is the principal curve of the *m*-th lineage at the branching point. The shrinkage process is defined as

$$c_{m}^{\mathrm{new}}\left(t\right)=w_{m}\left(t\right)c_{\mathrm{avg}}\left(t\right)+\left(1-w_{m}\left(t\right)\right)c_{m}\left(t\right),$$

where $w_{m}\left(t\right)$ is the weight of the *m*-th lineage. These modifications allow Slingshot to produce consistent pseudotime values across multiple lineages.

**Entropy-Based Pseudotime** One major challenge of the geodesic-based pseudotime is the appropriate determination of root cells, which often relies on prior biological knowledge. From a physical understanding, a cell’s pseudotime reflects the directionality of the underlying dynamical process, which the concept of entropy could quantify. Heuristically, higher entropy values typically indicate a more undifferentiated or pluripotent state where genes are more randomly expressed and the association between genes could be more prevalent. In comparison, lower entropy values suggest differentiated states, where the gene expression profile could be more concentrated on only a small number of pathways, and the gene interaction network could be more modular [47]. A proper entropy score based on such intuitions can thus be leveraged to estimate a cell’s relative position along developmental trajectories. Several methods have been developed from this perspective [48,49,50,51,52,53].

As a simple implementation, the entropy for one cell *i* is defined as [48]

$$H_{i}=-\sum_{j=1}^{d}p_{ij}\log p_{ij},$$

where $p_{ij}=\frac{X_{ij}}{N_{i}}$, and $X_{ij}$ represents the transcript count of gene *j* in cell *i*, and $N_{j}$ is the total transcript count for cell *i*.

One can extend this concept to a Markov chain model [50] to consider the interaction between the genes. Assume there is a predefined graph representing the gene–gene interaction (e.g., protein–protein interaction (PPI) network) from the existing database. The transition probability between genes *i* and *j* in cell *c* is

$$p_{ij}^{\left(c\right)}=\frac{x_{j}^{\left(c\right)}}{\sum_{k\in N(i)}x_{k}^{\left(c\right)}}=\frac{x_{j}^{\left(c\right)}}{{\left(Ax^{\left(c\right)}\right)}_{i}},$$

where $x_{i}^{\left(c\right)}$ is the expression level of gene *i* in cell *c*, N(i) are the neighbors of gene *i* in the graph, and A is the adjacency matrix of the graph. The corresponding stationary distribution is

$$\pi_{i}^{\left(c\right)}=\frac{x_{i}^{\left(c\right)}{\left(Ax^{\left(c\right)}\right)}_{i}}{x^{{\left(c\right)}^{T}}Ax^{\left(c\right)}},$$

and the Markov chain entropy (MCE) is defined as

$$\mathrm{MCE}=-\sum_{\left(i,j\right)\in \tilde{E}}\pi_{i}p_{ij}\log\left(\pi_{i}p_{ij}\right),$$

where $\tilde{E}$ includes all edges on the graph. The entropy of cell *c* is given by ${\mathrm{MCE}}^{\left(c\right)}$, computed using $\pi_{i}^{\left(c\right)}$ and $p_{ij}^{\left(c\right)}$. To determine the weights, ref. [51] proposes to optimize interaction weights based on cell expression $\pi^{\left(c\right)}=\frac{x^{\left(c\right)}}{∥x^{\left(c\right)}{∥}_{L_{1}}}$. For each cell, its MCE is maximized by solving

$$\max_{p_{ij}^{\left(c\right)}\geq 0}-\sum_{\left(i,j\right)\in \bar{E}}\pi_{i}^{\left(c\right)}p_{ij}^{\left(c\right)}\log\left(\pi_{i}^{\left(c\right)}p_{ij}^{\left(c\right)}\right),$$

subject to $\sum_{j\in N(i)}p_{ij}^{\left(c\right)}=1\;\mathrm{and}\;\sum_{i\in N(j)}\pi_{i}^{\left(c\right)}p_{ij}^{\left(c\right)}=\pi_{j}^{\left(c\right)}.$

#### 3.1.2. Discrete Dynamics Modeling

**Diffusion Pseudotime** Previous pseudotime methods, such as those based on geodesic paths or simple assumptions of pseudotime, typically lacked an underlying dynamical model to explain cell state transitions. These methods often relied on the assumption of continuous trajectories without explicitly modeling the stochastic processes driving those transitions. In contrast, methods like **DPT** [54] and **PAGA** [55] introduce stochastic dynamics through Markov chains. While still estimating pseudotime, these methods incorporate random walk-defined observed samples of single cells, allowing for a more quantitative treatment of how cell states evolve over time, with transitions captured probabilistically. As a result, they provide a more mechanistic approach to pseudotime estimation, making them a natural progression from traditional geodesic-based methods.

Motivated by the DiffusionMap [56] algorithm for dimensionality reduction, DPT constructs a Markov chain between cells, defines a distance metric, and uses this distance as pseudotime. The transition probability of cell *i* moving to cell *j* is computed using a simple Gaussian kernel, defined as

$$T_{ij}=\frac{1}{Z}W_{ij}=\frac{1}{Z}\left(\frac{2\sigma_{i}\sigma_{j}}{\sigma_{i}^{2}+\sigma_{j}^{2}}\right)\exp\left(-\frac{∥x_{i}-x_{j}{∥}^{2}}{2(\sigma_{i}^{2}+\sigma_{j}^{2})}\right),$$

where $Z_{i}=\sum_{j\in N(i)}W_{ij}$ is the normalization factor, and the hyperparameters $\sigma_{i},\sigma_{j}$ are the Gaussian kernel widths for cell *i* and cell *j*. DPT assumes that the distance in the eigenspace of the transition matrix *T* is related to the pseudotime ordering of cells. After removing steady-state eigenspace, the system’s dynamics are captured by the transition matrix $\bar{T}=T-\psi_{1}\psi_{1}^{T}$, where $\psi_{1}$ represent the eigenvector corresponding to the largest eigenvalue of the transition matrix. The dynamics are analyzed by summing all *t*-step transition matrices to compute the cumulative probability of state transitions across multiple walk lengths

$$M=\sum_{t=1}^{\infty }{\bar{T}}^{t}={\left(1-\bar{T}\right)}^{-1}-I.$$

Using this matrix M, a new distance metric is defined as

$${\mathrm{dpt}}^{2}\left(i,j\right)=∥M_{i,\cdot }-M_{j,\cdot }∥=\sum_{k=2}^{n}{\left(\frac{\lambda_{k}}{1-\lambda_{k}}\right)}^{2}{\left(\psi_{k}\left(i\right)-\psi_{k}\left(j\right)\right)}^{2},$$

where $\psi_{k}\left(i\right)$ represents the *i*-th component of the eigenvector corresponding to the *k*-th largest eigenvalue of the transition matrix. This metric simultaneously captures both short-range and long-range cell state transitions, making it useful for understanding the trajectory of cell states over time.

PAGA generalizes the DPT distance metric to disconnected graphs to deal with the existence of multiple distinct lineages in the dataset. In PAGA, graph construction begins by reducing the dimensionality of the gene expression data using PCA, followed by the construction of a KNN graph where the nodes represent cells. The graph is then partitioned into cell clusters using the Louvain algorithm, reminiscent of the **attractors** concept in the random walk. Two groups are considered connected if the actual number of edges $\epsilon_{ij}$ between them significantly exceeds the expected number of edges. The DPT distance metric is then extended to the disconnected graph. In practice, one treats cells that belong to separate clusters as being at an infinite distance from each other. For cells within the same connected region, one calculates distances between them similar to the calculation in DPT. This modification allows PAGA to estimate pseudotime and infer trajectories even in the presence of disconnected or sparse data regions.

**Random Walk with Directionality** Building on the inferred DPT, Palantir [57] introduces directionality into the cellular random walk, which is further developed in [58,59]. One simple approach is to prune the weight matrix as follows:

$${\bar{W}}_{ij}=\left\{\begin{array}{cc} W_{ij} & \mathrm{if}\;t_{i}\leq t_{j}\;\mathrm{or}\;0<t_{i}-t_{j}<\sigma_{i}\\ 0 & \mathrm{if}\;t_{i}-t_{j}>\sigma_{i} \end{array}\right.$$

For the directional Markov chain induced by the weight matrix $\bar{W}$, terminal states can be determined from its stationary distribution. Using absorption Markov chain theory, a cell fate matrix F can be derived from the canonical form of transition probability matrix in Equation (1), where $F={\left(I-Q\right)}^{-1}S$. Here, the element $F_{ij}$ represents the probability that a random walk starting from transient cell *i* will eventually be absorbed by terminal cell *j*. Specifically, the fate vector $f_{i}$ of a transient cell *i* corresponds to the *i*-th row of F, capturing the probability of differentiation into various states. To quantify the differentiation potential of a cell, one can then determine the entropy of the fate vector, or the Kullback–Leibler (KL) divergence between the fate vector $f_{i}$ and the average fate vector $\bar{f}$. Author: Please check that the intended meaning has been retained.

Another method to define the directional random walk on cells is Population Balance Analysis (PBA) [60]. Let G be the *k*-nearest neighbor graph of {X} and L its graph Laplacian. A potential function is defined by $V=\frac{1}{2}L_{N}^{†}R$, where $L_{N}^{†}$ denotes the pseudo-inverse of $L_{N}$ and R is the estimated cell population production rate vector at each node, using the gene expression of predefined lists of proliferation relevant genes. The transition probabilities of this Markov chain are then directed by the potential function

$$P_{ij}=\left\{\begin{array}{cc} \exp\left(\frac{V_{i}-V_{j}}{D}\right)\; & \mathrm{if}\;\left(i,j\right)\;\mathrm{is}\;\mathrm{in}\;G_{N}\\ 0 & \mathrm{otherwise} \end{array}\right.$$

After the random walk is constructed, PBA utilizes the conditional mean first passage time to quantify the difference of pseudotime for any pair of transient cells *i* and *j*.

**Dissecting Dynamical Structure** While previous methods such as DPT, Palantir, and PBA construct the random walk dynamics on snapshots of individual cells, methods like **MuTrans** [61] and **CellRank** [62,63] take a deeper look into the dynamical structure of the system itself, especially focusing on **metastability** and **attractor** structures, therefore robustly dissecting the system’s latent dynamics to identify long-term patterns of the cell-state transition.

MuTrans [61] adopts a multi-scale reduction technique for a diffusion-based, unidirectional cellular random walk, to infer stable and transient cells from snapshot scRNA-seq. Central to MuTrans is a membership matrix $\chi_{i,k}$ representing the soft clustering probability that cell *i* belongs to attractor $S_{k},k=1,...,K$, which could be interpreted as a cell cluster. A transient cell might have multiple positive components in its attractor membership, while the distribution of stable cells tends to be concentrated in one specific attractor. Meanwhile, MuTrans also reduces the dynamics on the attractor level, using $P^{\left(\mathrm{coar}\right)}\in R^{K\times K}$ to represent the coarsened transition matrix, and $\pi^{\left(\mathrm{coar}\right)}$ to represent the stationary distribution of the coarse-grained Markov chain. The original cell-to-cell dynamics can then be reconstructed from the coarsened dynamics, and the transition probability between cells *i* and *j* is given by

$${\hat{P}}_{i,j}=\sum_{m,n=1}^{K}\chi_{i,m}P_{m,n}^{\left(\mathrm{coar}\right)}\chi_{j,n}\frac{\pi_{j}}{\pi_{n}^{\left(\mathrm{coar}\right)}}.$$

The goal is to minimize the discrepancy between the reconstructed cell–cell dynamics $\hat{P}$ and the actual dynamics P, which is achieved by minimizing $∥\hat{P}{-P∥}^{2}$. This can be done using an EM-like algorithm, alternately optimizing the elements of $P^{\left(\mathrm{coar}\right)}$ and $\chi_{i,j}$. With the inferred attractor membership matrix and coarse-grained transition probabilities among clusters, MuTrans then constructs a dynamical manifold inspired by the energy landscape concept [64] to visualize the transient and stable cells, and uses transition path theory [65] based on $P^{\left(\mathrm{coar}\right)}$ to calculate the most probable transition paths among attractors.

CellRank extends the analysis by introducing a coarse-graining strategy for directed cellular random walks, such as those induced by pseudotime [57] or RNA velocity as described in Section 5.1. The approach begins with the clustering of cells into macro-states (i.e., attractors) using the GPCCA (Generalized Perron Cluster Cluster Analysis) algorithm [66], which is based on the Shur decomposition of the directed transition matrix P. The membership matrix χ and the coarse-grained transition probability matrix between attractors $P^{\left(\mathrm{coar}\right)}$ can be computed based on the decomposition. Once the attractors are identified, the terminal states can be determined in which the diagonal elements of the coarse transition matrix $P^{\left(\mathrm{coar}\right)}$ exceed a certain threshold. Cells in terminal states can then be treated as absorption sets of the random walk, and the cell fate vector could be computed similarly to Palantir. 

#### 3.1.3. Continuous Dynamics Modeling

**RNA Velocity Model and Parameter Estimation** Based on the unspliced RNA and spliced RNA counts $u_{g}$ and $s_{g}$ for each gene, an underlying ODE model could be naturally derived based on mass-action law such that

(4) $$\begin{array}{cc} \frac{du_{g}}{dt} & =\alpha_{g}\left(t\right)-\beta_{g}\left(t\right)u_{g}\left(t\right),\\ \frac{ds_{g}}{dt} & =\beta_{g}\left(t\right)u_{g}\left(t\right)-\gamma_{g}\left(t\right)s_{g}\left(t\right), \end{array}$$

where $\alpha_{g}\left(t\right)$, $\beta_{g}\left(t\right)$, and $\gamma_{g}\left(t\right)$ represent the rates of mRNA transcription, splicing, and degradation, respectively. Here, $v_{g}=\frac{ds_{g}}{dt}$ is defined as the **RNA velocity** of gene *g* [26]. By concatenating the RNA velocities of all genes in a cell, a vector $v=(v_{1},v_{2},\cdots ,v_{n})$ is formed, which contains information about how the amounts of spliced RNA in the cell are changing. This vector represents the potential direction of the cell state evolution and can be used for downstream tasks of cell fate inference.

From an algorithm perspective, the central issue in the RNA velocity is to determine the parameters of the Equation (4) from static snapshot data, where the time *t* of each cell is not explicitly known. In the following sections, we will summarize methods for solving $v_{g}$ and downstream tasks that utilize $v_{g}$.

##### Steady-State Assumption: Parameter Estimation in Velocyto [26]

In the original RNA velocity paper [26], parameter estimation was performed using linear regression with steady-state assumption to avoid the reliance on latent time *t*. Firstly, it assumes that for all genes *g*, $\alpha_{g}\left(t\right)$, $\beta_{g}\left(t\right)$, and $\gamma_{g}\left(t\right)$ are time-invariant. Secondly, it assumes that all genes share the same splicing rate β. Denote $\tilde{\alpha }=\frac{\alpha }{\beta }$ and $\tilde{\gamma }=\frac{\gamma }{\beta }$. To compute RNA velocity, one only needs to estimate ${\tilde{\gamma }}_{g}$ with the steady-state assumption that $\frac{ds_{g}}{dt}=0$. Indeed, we have the linear relation ${\tilde{\gamma }}_{g}=\frac{u_{g}\left(t\right)}{s_{g}\left(t\right)},$ suggesting that under the steady-state assumption, ${\tilde{\gamma }}_{g}$ can be estimated using linear regression. In practice, since most cells do not satisfy this assumption, it is commonly assumed that cells in the upper-right or lower-left regions of a scatter plot with unspliced RNA on the x-axis and spliced RNA on the y-axis are in equilibrium. Therefore, the common algorithm implementation is to limit the linear regression to the top or bottom 5% of cells based on unspliced and spliced RNA levels.

If the dynamic equations Equation (4) are expressed probabilistically, the parameter estimation could be enhanced based on the linear regression formulation of steady-state stochastic model [30]. The equation could be expressed as the regression problem

$$\left[\begin{array}{c} \langle u_{g}\left(t\right)\rangle \\ \langle u_{g}\left(t\right)\rangle +2\langle u_{g}\left(t\right)s_{g}\left(t\right)\rangle \end{array}\right]={\tilde{\gamma }}_{g}\left[\begin{array}{c} \langle s_{g}\left(t\right)\rangle \\ 2\langle s_{g}^{2}\left(t\right)\rangle -\langle s_{g}\left(t\right)\rangle \end{array}\right]+\epsilon ,$$

where 〈x〉 denotes the expectation of random variable *x*. The regression equation incorporates both first-order and second-order moment information of $u_{g}\left(t\right)$ and $s_{g}\left(t\right)$ and can be solved using generalized least squares.

##### Dynamic Inference: Parameter Estimation in scVelo [30]

A major issue with steady-state analysis is that many transient cells would be discarded in the parameter estimation. The scVelo approach [30] circumvents the issues through the estimation of kinetic parameters ($\alpha_{g}$, $\beta_{g}$, $\gamma_{g}$) via an expectation-maximization (EM) algorithm by modeling all the cells with the dynamical process.

Guided by transcriptional regulation principles, **scVelo** models gene expression dynamics through two distinct transcriptional phases: (1) an *induction phase* (k=0) characterized by promoter activation and transcriptional upregulation, and (2) a subsequent *repression phase* (k=1) marked by transcriptional suppression. This phase-specific regulation manifests through different unspliced RNA production rates ($\alpha_{g}^{\left(0\right)}\neq \alpha_{g}^{\left(1\right)}$). Let $t_{g}^{\left(k\right)}$ denote the transition time from phase k−1 to *k* for gene *g*, with initial conditions $u_{g}^{★}=u_{g}\left(t_{g}^{\left(k\right)}\right)$ and $s_{g}^{★}=s_{g}\left(t_{g}^{\left(k\right)}\right)$. The analytical solution to Equation (4) during phase *k* yields

$$\begin{array}{ccc} u_{g}\left(t\right) & =u_{g}^{★}e^{-\beta_{g}\tau }+\frac{\alpha_{g}^{\left(k\right)}}{\beta_{g}}\left(1-e^{-\beta_{g}\tau }\right),\\ s_{g}\left(t\right) & =s_{g}^{★}e^{-\gamma_{g}\tau }+\frac{\alpha_{g}^{\left(k\right)}}{\gamma_{g}}\left(1-e^{-\gamma_{g}\tau }\right)+\frac{\alpha_{g}^{\left(k\right)}-\beta_{g}u_{g}^{★}}{\gamma_{g}-\beta_{g}}\left(e^{-\gamma_{g}\tau }-e^{-\beta_{g}\tau }\right),\tau & =t-t_{g}^{\left(k\right)}. \end{array}$$

For each gene *g*, one can estimate the parameter set $\theta_{g}=\left\{\alpha_{g}^{\left(k\right)},\beta_{g},\gamma_{g},t_{g}^{\left(k\right)}\right\}$ by minimizing the discrepancy between modeled trajectories ${\hat{x}}_{g}\left(t\right)=\left(u_{g}\left(t\right),s_{g}\left(t\right)\right)$ and observed single-cell measurements $x_{g,c}=\left(u_{g,c},s_{g,c}\right)$ across cells *c*. Assuming Gaussian residuals $e_{c}=∥x_{g,c}-{\hat{x}}_{g}\left(t_{c}\right)∥$ with variance $\sigma^{2}$, the log-likelihood function becomes

(5) $$\max_{\theta_{g},t_{c}}L\left(\theta_{g},,t_{c}\right)=-\frac{1}{2\sigma^{2}}\sum_{c}{∥x_{g,c}-{\hat{x}}_{g}\left(t_{c}\right)∥}^{2}+\mathrm{constant}.$$

The EM implementation proceeds as follows:

- **Initialization:** Using steady-state estimation as the initial value for iteration.
  $$\begin{array}{cc} \beta_{g} & =1,\;\gamma_{g}=\frac{u_{g}^{⊤}s_{g}}{∥s_{g}{∥}^{2}},\\ k_{g,c} & =I\left(u_{g,c}-\tilde{\gamma }s_{g,c}\geq 0\right),\;\alpha_{g}^{\left(1\right)}=\max_{c}s_{g,c},\;\alpha_{g}^{\left(0\right)}=0. \end{array}$$
- **E-step:** Assigning hidden latent time $t_{c}$ for each cell by projecting observations onto the current estimated trajectory ${\hat{x}}_{g}\left(t|\theta_{g}\right)$.
- **M-step:** Updating $\theta_{g}$ via maximum likelihood estimation given current latent time assignments.

##### Function Class-Based Estimation

While traditional RNA velocity methods focus on estimating parameters $\alpha_{g},\beta_{g},\gamma_{g}$ in dynamic Equation (4), alternative approaches such as UniTVelo [67] and TF Velo [68] take a different path by directly parameterizing the dynamics of spliced RNA. UniTVelo [67] models transcriptional phases through radial basis functions

$$\begin{array}{cc} s_{g}\left(t_{g,c}\right) & =h_{g}\exp\left(-a_{g}{\left(t_{g,c}-\tau_{g}\right)}^{2}\right)+o_{g},\\ u_{g}\left(t_{g,c}\right) & =\frac{1}{\beta_{g}}\left({\dot{s}}_{g}\left(t_{g,c}\right)+\gamma_{g}s_{g}\left(t_{g,c}\right)\right)+i_{g}, \end{array}$$

where the velocity derives directly from function differentiation ${\dot{s}}_{g}\left(t_{g,c}\right)=-2a_{g}\left(t_{g,c}-\tau_{g}\right)s_{g}\left(t_{g,c}\right).$ The full parameter set $\left(h_{g},a_{g},\tau_{g},o_{g},\gamma_{g},\beta_{g},i_{g},t_{g,c}\right)$ is estimated via maximum likelihood framework as Equation (5), comparing model predictions ${\hat{x}}_{c}=\left(u_{g}\left(t_{g,c}\right),s_{g}\left(t_{g,c}\right)\right)$ against observations $x_{c}=\left(u_{g,c},s_{g,c}\right)$ under Gaussian residuals.

TF Velo [68] introduces transcription factor coupling through linear dynamics ${\dot{s}}_{g}\left(t\right)=w_{g}^{⊤}f_{g}\left(t\right)-\gamma_{g}s_{g}\left(t\right),$ combined with sinusoidal splicing $s_{g}\left(t\right)=A_{g}\sin\left(\omega_{g}t+\theta_{g}\right)+b_{g}.$ This functional specification enables the analytical resolution of TF interactions

$$w_{g}^{⊤}f_{g}\left(t\right)=A_{g}\sqrt{4\pi^{2}+\gamma_{g}^{2}}\sin\left(2\pi t+\theta_{g}+\phi_{g}\right)+b_{g}\gamma_{g},\;\phi_{g}=\arctan\left(2\pi /\gamma_{g}\right).$$

Parameters are optimized by matching predicted trajectories ${\hat{x}}_{c}=\left(w_{g}^{⊤}f_{g}\left(t_{g,c}\right),s_{g}\left(t_{g,c}\right)\right)$ to observed data $x_{c}=\left(w_{g}^{⊤}f_{g,c},s_{g,c}\right)$ using the same likelihood framework as Equation (5).

**Deep Learning-Based RNA Velocity** Recently, the application of deep learning methods has expanded the possibilities for RNA velocity estimation [69,70,71]. In RNA velocity analysis, the expressive power of neural networks is especially useful in inferring the latent state of cells, as well as encouraging the consistency of the learned vector field.

##### Latent State: VAE-Based Methods

The Variational Autoencoder (VAE) [72] is an effective approach to model the distribution of data through latent variables to achieve high-dimensional data reconstruction. Its core idea is to introduce a latent variable z and express the distribution of data x as the following conditional distribution: p(x)=∫p(x|z)p(z)dz, where the generative process is z∼p(z),x∼p(x|z). The VAE employs a decoder $p_{\theta }\left(x|z\right)$ and an encoder $q_{\phi }\left(z|x\right)$ to map between the latent space and data space. The training objective is the Evidence Lower Bound (ELBO):

$$L_{\mathrm{ELBO}}=L_{\mathrm{rec}}+L_{\mathrm{reg}}=E_{q_{\phi }\left(z|x\right)}\left[\log p_{\theta }\left(x|z\right)\right]-D_{\mathrm{KL}}\left[q_{\phi }\left(z|x\right)∥p\left(z\right)\right],\;x∼p_{\mathrm{data}}\left(x\right),$$

where the first term is the **reconstruction loss**, enforcing similarity between decoded data and observations, and the second term acts as a **regularizer**, aligning the latent distribution with the prior. Typically, the decoder outputs the mean $\mu_{\theta }\left(z\right)$ of a Gaussian distribution with fixed variance $\sigma^{2}$, leading to $L_{\mathrm{rec}}\propto -\frac{1}{2\sigma^{2}}E_{q_{\phi }\left(z|x\right)}\left[∥x-\mu_{\theta }{\left(z\right)∥}^{2}\right].$ Several methods are based on VAE to improve the RNA velocity model by taking advantage of the latent space.

VeloAE [73] computes RNA velocity using latent space representations. Its encoder maps the spliced RNA matrix $S\in R^{n_{c}\times n_{g}}$ and unspliced RNA matrix $U\in R^{n_{c}\times n_{g}}$ to latent representations $\tilde{S}\in R^{n_{c}\times d_{z}}$ and $\tilde{U}\in R^{n_{c}\times d_{z}}$, while the decoder reconstructs $\hat{S}$ and $\hat{U}$. Velo AE enforces the steady-state constraint on latent representations ${\tilde{u}}_{i},{\tilde{s}}_{i}$, resulting in a composite loss:

$$L=L_{\mathrm{rec}}+L_{\mathrm{reg}}=\sum_{i=1}^{d_{z}}\mathrm{MSE}\left({\tilde{u}}_{i}-\gamma_{i}{\tilde{s}}_{i}\right)+\left[\mathrm{MSE}\left(S,\hat{S}\right)+\mathrm{MSE}\left(U,\hat{U}\right)\right].$$

The RNA velocity is derived as $\tilde{u_{i}}-\gamma_{i}\tilde{s_{i}}$ after training.

LatentVelo [74] incorporates latent variables $z_{c}=\left(u_{c}^{\left(z\right)},s_{c}^{\left(z\right)}\right)$ and pseudotime $t_{c}$, representing latent-space unspliced/spliced RNA levels and cellular pseudotime. It assumes the following dynamics:

$$\begin{array}{cc} \frac{du_{c}^{\left(z\right)}\left(t\right)}{dt} & =f_{u}\left(u_{c}^{\left(z\right)}\left(t\right),r_{c}^{\left(z\right)}\left(t\right)\right),\\ \frac{ds_{c}^{\left(z\right)}\left(t\right)}{dt} & =f_{s}\left(u_{c}^{\left(z\right)}\left(t\right),s_{c}^{\left(z\right)}\left(t\right)\right),\\ \frac{dr_{c}^{\left(z\right)}\left(t\right)}{dt} & =f_{r}\left(s_{c}^{\left(z\right)}\left(t\right),r_{c}^{\left(z\right)}\left(t\right),h_{c}\right),\\ h_{c} & =f_{h}\left(s_{c}^{(z),\mathrm{obs}},u_{c}^{(z),\mathrm{obs}}\right), \end{array}$$

where $s_{c}^{(z),\mathrm{obs}}$ and $u_{c}^{(z),\mathrm{obs}}$ are latent-space observations, $r_{c}^{\left(z\right)}$ governs chromatin dynamics, and $f_{u},f_{s},f_{r},f_{h}$ are neural networks with $f_{h}$ computing the cell state encoding $h_{c}$. Beyond standard VAE losses, an **evolution loss** ensures correct dynamics:

$$L_{\mathrm{evol}}=\sum_{c=1}^{n_{c}}E_{t_{c}∼q\left(t_{c}|x\right)}\left[\frac{∥z_{c}^{\mathrm{obs}}-z_{c}\left(t_{c}\right){∥}^{2}+∥x_{c}-{\hat{x}}_{c}\left(t_{c}\right)∥}{\sigma^{2}}\right].$$

The total loss is $L=L_{\mathrm{rec}}+L_{\mathrm{reg}}+L_{\mathrm{evol}}$.

VeloVI [75] models gene-specific state distributions $\pi_{g,c}$ with a Dirichlet prior $\pi_{gc}∼\mathrm{Dirichlet}\left(\frac{1}{4},\frac{1}{4},\frac{1}{4},\frac{1}{4}\right)$, where states $k_{g,c}\in \left\{1,2,3,4\right\}$ correspond to induction, repression, induction steady, and repression steady. Key parameters include gene-specific rates $\alpha_{g},\beta_{g},\gamma_{g}$, pseudotime $t_{g,c}$, and switching time $t_{g}^{s}$. For repression-related states, $\alpha_{g}=0$ is fixed. Genes in transient states follow Equation (4), while steady states use analytic solutions. Reconstruction losses follow standard VAE training.

VeloVAE [76] uses latent variables $z_{c}∼N\left(0,I\right)$ and pseudotime $t_{c}∼N\left(t_{0},\sigma_{0}^{2}\right)$. A fully connected network maps z to gene-specific parameters $\alpha_{c,g},\beta_{c,g},\gamma_{c,g}$, enabling reconstruction via Equation (4) and training with standard VAE losses.

##### Enhancing Velocity: Continuity-Based Methods

Several methods leverage the **continuity assumption** in single-cell data: If the observed data fully capture the continuous dynamics of cellular evolution, a cell’s state at the next timestep should align with its neighbors. Several methods use such prior information for defining the loss function to further refine the RNA velocity.

DeepVelo [77] parameterizes $\alpha_{g,c},\beta_{g,c},\gamma_{g,c}$ via neural networks to compute RNA velocity $v_{g,c}$. Its loss function incorporates temporal consistency: The state $s_{c}\left(t+1\right)$ is approximated as a weighted sum of neighboring cell states $s_{j}\left(t\left(j\right)\right)$, where the transition probability $P_{+}\left(c\to j\right)$ is defined as

$$P_{+}\left(c\to j\right)=\left\{\begin{array}{cc} \frac{1}{Z} & \mathrm{if}\;\cos\left(s_{j}-s_{c},{\hat{v}}_{c}\right)>0\;\mathrm{and}\;j\in N\left(c\right),\\ 0 & \mathrm{otherwise}. \end{array}\right.$$

where *Z* is the normalization constant. The forward consistency loss enforces this assumption as follows:

$$L_{+}=\sum_{c=1}^{n_{c}}{∥s_{c}\left(t\right)+{\hat{v}}_{c}-s_{c}\left(t+1\right)∥}^{2},$$

with $s_{c}\left(t+1\right)=\sum_{j\in N(c)}s_{j}\left(t\left(j\right)\right)P_{+}\left(c\to j\right)$. A symmetric backward consistency loss $L_{-}$ is similarly constructed. Additionally, a correlation loss ensures consistency with transcriptional dynamics in the following way:

$$L_{\mathrm{corr}}=-\left[\lambda_{u}\frac{{\hat{v}}_{c}\cdot u_{c}}{∥{\hat{v}}_{c}∥∥u_{c}∥}+\lambda_{s}\frac{{\hat{v}}_{c}\cdot s_{c}}{∥{\hat{v}}_{c}∥∥s_{c}∥}\right],$$

yielding the total loss $L=L_{+}+L_{-}+L_{\mathrm{corr}}$.

CellDancer [78] similarly parameterizes $\alpha_{g,c},\beta_{g,c},\gamma_{g,c}$ with neural networks, and estimates velocity as

$${\hat{v}}_{c}=\left(\Delta u_{c},\Delta s_{c}\right),\;\mathrm{where}\;\Delta u_{c}=\alpha_{c}-\beta_{c}⊙u_{c},\;\Delta s_{c}=\beta_{c}-\gamma_{c}⊙s_{c}.$$

Its loss maximizes velocity alignment with neighbors, as follows:

$$L=\sum_{c}\left[1-\max_{j\in N(c)}\cos\left({\hat{v}}_{c},v_{j}\right)\right],\;v_{j}=\left(u_{j}-u_{c},s_{j}-s_{c}\right).$$

**Vector Field Reconstruction Based on RNA Velocity** After estimating the model parameters and obtaining the RNA velocity for each cell, an important downstream task is to predict the fate of cells based on the estimated velocity, e.g., the continuous differentiation trajectory of cells. To construct a continuous vector field, the RNA velocity of each cell could be treated as the value of a vector field at discrete points. Then, the vector field is reconstructed by formulating a regression problem. Subsequently, quantities derived from vector field analysis, such as equilibrium points, streamlines, gradients, divergence, and curl, are used to study cell fate in continuous dynamics setup [79].

##### Estimating the Vector Field

Denote the spliced RNA counts and RNA velocities at various data points ${\left\{x_{i},v_{i}\right\}}_{i=1}^{n}$. The task is to find a continuous vector field function $f^{★}$ that minimizes the regression loss $L=\sum_{i}p_{i}{∥v_{i}-f\left(x_{i}\right)∥}^{2}$ where $p_{i}$ denotes the weights of each data point.

Dynamo [79] approximates the unknown vector-valued function in a sparse reproducing kernel Hilbert space (RKHS). For a vector-valued function f∈H in RKHS, it can be represented as a sum of Gaussian kernels as follows:

$$f\left(x\right)=\sum_{i=1}^{m}\Gamma \left(x,{\tilde{x}}_{i}\right)c_{i},\;\Gamma \left(x,\tilde{x}\right)=\exp(-w∥x-\tilde{x}{∥}^{2}),$$

where ${\tilde{x}}_{i}$ are called control points. Additionally, the norm of f in H can be computed as ${∥f∥}^{2}=\sum_{i,j=1}^{m}c_{i}^{T}\Gamma \left({\tilde{x}}_{i},{\tilde{x}}_{j}\right)c_{j}^{T}.$ The loss function for the vector field estimation problem includes a regularization term based on the norm of f such that $L_{\lambda }=\sum_{i}p_{i}∥v_{i}-f\left(x_{i}\right){∥}^{2}+\frac{\lambda }{2}{∥f∥}^{2},$ where λ is the regularization coefficient.

Another popular fitting strategy to reconstruct the continuous vector field is to use the neural network, where a VAE-based deep learning method was proposed in [80].

##### Geometric Analysis of Vector Field

Based on the estimated continuous vector field, Dynamo [79] proposed several analyses to reveal the differential geometry of the RNA velocity. First, the Jacobian matrix is essential for analyzing the stability of equilibrium points in a dynamical system and for studying gene–gene interactions. In RKHS context, the Jacobian matrix can be analytically computed as

$$J=\frac{\partial f(x)}{\partial x}=-2w\sum_{i=1}^{m}\Gamma \left(x,{\tilde{x}}_{i}\right)c_{i}{\left(x-{\tilde{x}}_{i}\right)}^{T}$$

where $\Gamma (x,{\tilde{x}}_{i})$ is the Gaussian kernel. Since the (i,j)-th entry of the Jacobian matrix $J_{ij}$ represents the effect of unspliced RNA levels of gene *j* on the RNA velocity of gene *i*, the Jacobian matrix can be used to analyze the strength of gene–gene interactions. By averaging the Jacobian matrix across all data points, an average Jacobian matrix 〈J〉 can be obtained. By sorting the elements in each row of 〈J〉, the top regulators for each effector can be identified. Conversely, by sorting the elements in each column of 〈J〉, the top effectors for each regulator can be identified. The Jacobian matrix can also be used to compute the effect of perturbations. If the system state changes by Δx at x, the resulting change in the vector field is Δf=JΔx.

Several other quantities could also be conveniently derived based on the Jacobian matrix.

- The divergence represents the net flux generated or dissipated per unit time at each point in the vector field:
  ∇·f=Tr(J),
  where Tr(J) denotes the trace of the Jacobian matrix. Regions with divergence greater than 0 (sources) may correspond to the initial states of cells, while regions with divergence less than 0 (sinks) may correspond to the terminal states of cells.
- The acceleration of a particle moving along the streamlines of the vector field can be directly computed from the Jacobian matrix:
  $$a=\frac{dv}{dt}=Jv.$$
- The curvature vector of the streamlines is defined as the derivative of the unit tangent vector with respect to time:
  $$\kappa =\frac{1}{∥v∥}\frac{d}{dt}\frac{v}{∥v∥}=\frac{\left(v^{T}v\right)Jv-\left(v^{T}Jv\right)v}{{∥v∥}^{4}}.$$

##### Transition Path Analysis

Based on the learned vector field, continuous cell trajectories could also be constructed in Dynamo [79] using the concept of most probable path [64]. Given the SDE (2), the action along any path Ψ is defined as

$$S_{T}\left[\psi \right]=\int_{0}^{T}L^{FW}\left(\psi ,\dot{\psi }\right)dt,L^{FW}\left(\psi ,\dot{\psi }\right)=\frac{1}{4}{\left[\dot{\psi }\left(s\right)-b\left(\psi (s)\right)\right]}^{t}D^{-1}\left(\psi (s)\right)\left[\dot{\psi }\left(s\right)-b\left(\psi (s)\right).\right]$$

According to the Freidlin–Wentzell theorem [64], the path of least action is indeed the most probable path to make transitions between two attractors. In actual computation, Dynamo assumes the constant noise coefficient by taking $D=\frac{\sigma^{2}}{2}$ since only a continuous vector field is reconstructed.

For a transition connecting two meta-stable states along the optimal path with action $S^{★}\geq 0$, the transition rate between the attractors is given by

(6) $$R\left(x_{s}\to x_{t}\right)\approx C\exp\left(-S^{★}\right),$$

where *C* is a proportionality constant.

### 3.2. Temporally Resolved Single-Cell RNA-Seq

**Figure 3:**
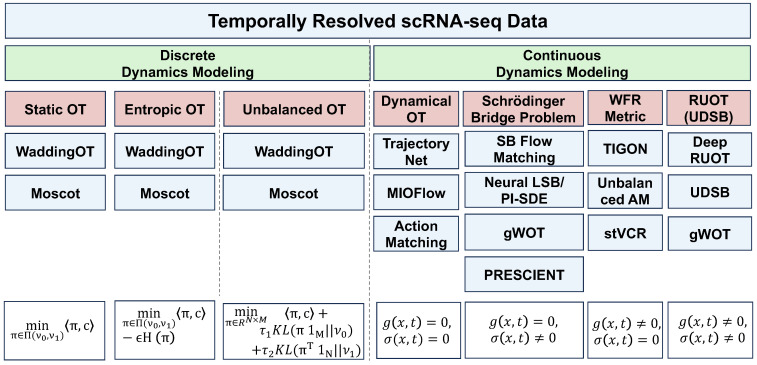
**Dynamic modeling of temporally-resolved single-cell transcriptomics**.

Temporally resolved scRNA-seq provides us with a deeper understanding of the dynamics process in single cells. However, due to the destructive nature of scRNA-seq technology, we cannot track the trajectories of individual cells. Instead, we can only observe the changes in cellular distribution with time. Thus, reconstructing the trajectories of single cells from samples collected at discrete and sparse temporal points becomes crucial for understanding developmental processes and other dynamic biological processes and remains a challenging problem [13,35,41,81,82,83,84,85,86,87,88,89,90,91]. To overcome these challenges, many methods have been developed in recent years. From a dynamical perspective, these approaches can be broadly classified into two categories: those that model dynamics on discrete cell states and those that model dynamics in continuous spaces. In the following, we will introduce these methods separately from these two viewpoints. Figure 3 summarizes these approaches.

#### 3.2.1. Discrete Temporal Dynamics Modeling

Among the methods that model dynamics on discrete cell states, pioneering work includes Waddington OT [35] and Moscot [36]. These approaches employ static optimal transport as the main tools.

**Static Optimal Transport** To formulate this problem, consider $X\in R^{N\times G}$ and $Y\in R^{M\times G}$ represent two unpaired datasets of *N* and *M* cells observed at different time points ($t_{1},t_{2}$), respectively, in the *G* dimensional gene expression space. Then, one can define two marginal distributions $\nu_{0}\in C_{N}$, $\nu_{1}\in C_{M}$ at $t_{1}$ and $t_{2}$ respectively on the probability simplex $C_{N}=\left\{a\in R^{N}|\sum_{i=1}^{N}a_{i}=1,a\geq 0\right\}.$ The goal of optimal transport is to find the optimal coupling $\pi \in R_{+}^{N\times M}$ that transports a distribution to another while minimizing the cost associated with the transportation. The feasible transport plan is defined as $\Pi \left(\nu_{0},\nu_{1}\right)=\left\{\pi \in R^{N\times M}:\pi 1_{M}=\nu_{0},\pi^{T}1_{N}=\nu_{1},\pi \geq 0\right\}.$ So the static optimal transport problem is formally defined as

(7) $$\begin{array}{c} \min_{\pi \in \Pi (\nu_{0},\nu_{1})}\langle \pi ,c\rangle =\sum_{i,j}c_{i,j}\pi_{i,j}. \end{array}$$

The cost matrix $c\in R_{+}^{n\times m}$ defines the transportation cost between each pair of points, where $c_{ij}:=c\left(x_{i},y_{j}\right)$ quantifies the expense of transferring a unit mass from the source point $x_{i}$ to the target point $y_{j}$. By solving the static optimal transport problem, one can determine the transport matrix that couples the two distributions. This static optimal transport can be effectively addressed using the Python Optimal Transport (POT) library [92].

**Entropic Optimal Transport** Additionally, to enhance the efficiency of solving the optimal transport problem, a regularized optimal transport approach is often introduced. The discrete entropy of a coupling matrix is defined as

$$H\left(\pi \right)\overset{\;\mathrm{def}\;}{=}-\sum_{i,j}\pi_{i,j}\left(\log\left(\pi_{i,j}\right)-1\right).$$

The function *H* is 1 -strongly concave, because its Hessian is $\partial^{2}H\left(\pi \right)=-\mathrm{diag}\left(1/\pi_{i,j}\right)$ and $0<\pi_{i,j}\leq 1$. The idea of the entropic regularization of optimal transport is to use −H as a regularizing function to obtain approximate solutions to the original transport problem:

(8) $$\min_{\pi \in \Pi (\nu_{0},\nu_{1})}\langle \pi ,c\rangle -\varepsilon H\left(\pi \right)$$

Since the objective is an ε-strongly convex function, problem (8) has a unique optimal solution. Using the KKT conditions, the solution to (8) is unique and has the form [93]

$$\forall \left(i,j\right)\in \left(N\times M\right),\;\pi_{i,j}=a_{i}K_{i,j}b_{j}$$

for two unknown variables $\left(a,b\right)\in R_{+}^{N}\times R_{+}^{M}$, where $K_{i,j}=e^{-\frac{c_{i,j}}{\varepsilon }}$.

*Sinkhorn Algorithm* From above, the optimal solution of problem (8) can be expressed in matrix form as π=diag(a)Kdiag(b). Then it is necessary to satisfy the constraints $\Pi (\nu_{0},\nu_{1})$, i.e.,

$$\mathrm{diag}\left(a\right)K\mathrm{diag}\left(b\right)1_{M}=\nu_{0},\;\mathrm{diag}\left(a\right)K^{T}\mathrm{diag}\left(b\right)1_{N}=\nu_{1}.$$

Note that $\mathrm{diag}\left(b\right)1_{M}$ is b and the multiplication of diag(a) times Kb is

$$a⊙\left(Kb\right)=\nu_{0},a⊙\left(K^{T}b\right)=\nu_{1},$$

where ⊙ represents the entry-wise multiplication. An intuitive way is to solve them iteratively, and these two updates yield the Sinkhorn algorithm,

$$a^{\left(\ell +1\right)}\overset{\;\mathrm{def}.\;}{=}\frac{\nu_{0}}{Kb^{\left(\ell \right)}},\;\;b^{\left(\ell +1\right)}\overset{\;\mathrm{def}.\;}{=}\frac{\nu_{1}}{K^{T}a^{\left(\ell +1\right)}}.$$

The division used above between two vectors is entry-wise and can be computed in time and memory quadratically of cell number.

**Unbalanced Optimal Transport** Static optimal transport inherently conserves mass. However, during cellular development and differentiation, processes such as cell proliferation and apoptosis result in mass non-conservation. Consequently, it is essential to consider unnormalized distributions that account for cell growth and death. Additionally, the marginals must be adjusted to incorporate these factors effectively. So, the unbalanced optimal transport can be defined as follows:

(9) $$\min_{\pi \in R_{+}^{N\times M}}\langle \pi ,c\rangle +\tau_{1}\mathrm{KL}(\pi 1_{M}\left|\right|\nu_{0})+\tau_{2}\mathrm{KL}(\pi^{T}1_{N}\left|\right|\nu_{1}),$$

where $\tau_{1}$ and $\tau_{2}$ are hyperparameters that control the degree of penalization. When $\tau_{1}=\tau_{2}\to +\infty$, then one can recover the original optimal transport. To further adjust the marginal distributions accounting for growth and death [35,36], for the left marginal distribution $\nu_{0}$ we set

(10) $${\left(\nu_{0}\right)}_{i}=\frac{g{\left(x_{i}\right)}^{t_{2}-t_{1}}}{\sum_{j=1}^{N}g{\left(x_{j}\right)}^{t_{2}-t_{1}}},\;\forall i\in \left\{1,\ldots ,N\right\}.$$

where *g* is the growth/death function and can be estimated through the gene sets. For the right marginal distribution $\nu_{1}$, set it as the uniform distribution, i.e., ${\left(\nu_{1}\right)}_{j}=1/M,\;\forall j\in \left\{1,\ldots ,M\right\}.$

By the obtained coupling matrix $\pi \in R_{+}^{N\times M}$, one can perform biological downstream analysis such as computing ancestors or descends of a cell state and imputing gene expressions [35,36]. Naturally, a Markov chain model can be formulated to quantify the transition probability among cells across time points based on the optimal transport plan [36,63]. By weighting this random walk with those induced by other quantities such as gene expression similairity (e.g., DiffuionMap), pseudotime (e.g., Palantir), or RNA velocity, the CellRank analysis could also be conducted to dissect the underlying structure of the transitional dynamics [63].

#### 3.2.2. Continuous Temporal Dynamics Modeling

Although static optimal transport provides a robust framework for coupling distributions at different time points, there is a substantial interest in capturing continuous cellular dynamics over time and fitting mechanistic models that transform the source distribution into the target distribution. This interest has driven the development of various dynamical optimal transport (OT) methods and flow-based generative models. Prominent approaches include those based on the Benamou–Brenier formulation [94], such as TrajectoryNet [95], MIOFlow [96], and other related methodologies [90,97,98,99,100,101]. Additionally, unbalanced dynamic OT methods [41,81,86,89], Gromov–Wasserstein OT approaches [102], continuous normalizing flows (CNF), and conditional flow matching techniques (CFM) [37,38,103,104,105,106,107,108,109,110,111] have also been proposed. Despite these advancements, many of these methods do not fully account for stochastic dynamical effects, particularly the intrinsic noise inherent in gene expression and cell differentiation [61,112], which are prevalent in single-cell biological processes [113].

In the realm of stochastic dynamics, the Schrödinger bridge (SB) problem seeks to identify the most probable stochastic transition path between two arbitrary distributions relative to a reference stochastic process [114]. Variants of the SB problem have been applied across various domains, including single-cell RNA sequencing (scRNA-seq) analysis and generative modeling. These approaches encompass static methods [82,115,116,117,118,119,120,121,122], dynamic methods [84,88,91,123,124,125,126,127,128,129,130,131,132,133,134,135], and flow-matching techniques [136]. However, these methods often fail to address unnormalized distributions resulting from cell growth and death. To address this problem, some methods have been developed to account for the unbalanced stochastic dynamics, for example, those based on branching SDE theory (e.g, gWOT) [82,87,116,117] and those based on the Feynman–Kac formula with forward-backward SDE theory [115]. Among those, most methods often require prior knowledge of these processes, such as growth or death rates [82,87,115,117] or depend on additional information like cell lineage data [116].

Recently, regularized unbalanced optimal transport (RUOT), also known as unbalanced Schrödinger bridge [137], has emerged as a promising approach for modeling stochastic unbalanced continuous dynamics [137,138,139,140]. RUOT can be viewed as an unbalanced relaxation of the dynamic Schrödinger bridge formulation. For instance, ref. [138] elucidates the connection between certain RUOT formulations and branching Schrödinger bridges. Meanwhile, a new deep learning framework (DeepRUOT) [40], has been developed to learn general RUOT and infer continuous unbalanced stochastic dynamics from sample data based on derived Fisher regularization forms without requiring prior knowledge.

The primary objective now transforms to determine the dynamics described by Equations (2) and (3) from observed data, given unnormalized distributions at *T* discrete time points where $x_{i}\in R^{d}∼\nu_{i}$ for each fixed time point i∈{0,…,T−1}. Note that solely satisfying Equation (3) does not admit a unique solution. Consequently, we need to ensure that the inferred dynamics also adhere to certain energy minimization principles. Building upon Equation (3), the problem can be categorized into four distinct scenarios: (1) g(x,t)=0andσ(x,t)=0, (2) g(x,t)=0andσ(x,t)≠0, (3) g(x,t)≠0andσ(x,t)=0, (4) g(x,t)≠0andσ(x,t)≠0. Each of these cases is examined to systematically address the learning of the underlying dynamics.

**Dynamical Optimal Transport** **(**g=0,σ=0**)** In this case, it means the dynamics do not account for unblancedness and stochastic, i.e., the cellular dynamics are governed by $dx_{t}=b\left(x_{t},t\right)dt$. Then we can use the dynamical optimal transport to model these dynamics, also known as the Benamou–Brenier formulation [94], which can be stated as follows:

(11) $$\begin{array}{c} \frac{1}{2}W_{2}^{2}\left(\nu_{0},\nu_{1}\right)=\underset{\left(p(x,t),b(x,t)\right)}{\inf}\int_{0}^{1}\int_{R^{d}}\frac{1}{2}{∥b\left(x,t\right)∥}_{2}^{2}p\left(x,t\right)dxdt,\\ s.t.\partial_{t}p+\nabla \cdot \left(b(x,t)p\right)=0,\;p{{|}_{t=0}=\nu_{0},p|}_{t=1}=\nu_{1}. \end{array}$$

The inclusion of the factor $\frac{1}{2}$ on the left-hand side ensures that the Wasserstein distance has a more physically meaningful interpretation; for example, it represents the total action required to transport one distribution to another. In this formulation, probability distributions are connected through a deterministic transport equation. It has been demonstrated that this dynamic formulation is equivalent to static optimal transport problem (Equation (7)) when employing the cost function $c\left(x,y\right)={∥x-y∥}_{2}^{2}$.

##### Neural ODE Solver

Numerous methodologies have been proposed to solve dynamical optimal transport or its variants numerically. The basic approach involves employing a neural network, denoted as $b_{\theta }\left(x,t\right)$, to parameterize b(x,t), and subsequently utilizing the ordinary differential equations (ODEs) that govern particle trajectories. From Problem (11), it is evident that the optimization process must address two distinct loss components: the first pertains to the computation of an energy-related loss, while the second concerns the reconstruction error ($p\left(x,1\right)=\nu_{1}$).

Regarding energy loss, the high-dimensional nature of the integral presents significant challenges due to the curse of dimensionality. To mitigate this issue, the integral is approximated using Monte Carlo integration and continuous normalizing flow (CNF) techniques [95]. The strategy involves performing integration along the particle trajectories dictated by the ODE, i.e.,

$$\int_{0}^{1}\int_{R^{d}}\frac{1}{2}{∥b\left(x,t\right)∥}_{2}^{2}p\left(x,t\right)dxdt=E_{x_{0}∼\nu_{0}}\int_{0}^{1}\frac{1}{2}{∥b\left(x\left(t\right),t\right)∥}_{2}^{2}dt,$$

where x(t) satisfies the ODE $\frac{dx}{dt}=b\left(x,t\right),x_{0}∼\nu_{0}$. For the distribution reconstruction loss, the authors incorporate an additional penalizing constraint. By integrating these two loss components, there exists a sufficiently large λ≥0 such that [95,96]

$$\frac{1}{2}W_{2}^{2}\left(\nu_{0},\nu_{1}\right)=\underset{\left(p(x,t),b(x,t)\right)}{\inf}E_{x_{0}∼\nu_{0}}\int_{0}^{1}\frac{1}{2}{∥b\left(x\left(t\right),t\right)∥}_{2}^{2}dt+\lambda D\left(p\left(x,1\right),\nu_{1}\right).$$

Based on this formulation, TrajectoryNet [95] computes both the energy and the reconstruction errors by using neural ODE [141] to parametrize the velocity b(x,t).

##### Conditional Flow Matching

Recently, conditional flow matching (CFM) presents another efficient dynamical OT solver especially in high dimensionality case [37,38,104,129]. Assume that the probability path p(x,t) and the corresponding vector field b(x,t) generating it are known, and that p(x,t) can be efficiently sampled. Under these conditions, a neural network $b_{\theta }\left(x,t\right)$ can be trained to approximate b(x,t) by minimizing the flow matching (FM) objective:

$$L_{\mathrm{FM}}\left(\theta \right)=E_{t∼U(0,1),x∼p(x,t)}{∥b_{\theta }\left(x,t\right)-b\left(x,t\right)∥}_{2}^{2}.$$

However, this objective is computationally intractable when dealing with general source and target distributions. Consider the specific case of Gaussian marginal densities, defined as $p\left(x,t\right)=N(x|\mu \left(t\right),\sigma {\left(t\right)}^{2}I)$. The corresponding unique vector field that generates this density from $N(x|\mu \left(0\right),\sigma {\left(0\right)}^{2}I)$ is $b\left(x,t\right)=\mu^{\prime }\left(t\right)+\frac{\sigma (t)}{\sigma^{\prime }\left(t\right)}\left(x-\mu \left(t\right)\right),$ where $\mu^{\prime }\left(t\right)$ and $\sigma^{\prime }\left(t\right)$ means the time derivative [37,38]. Now, assume the marginal probability trajectory p(x,t) is a mixture of conditional probability paths p(x,t|z). Specifically, this can be expressed as:

p(x,t)=∫p(x,t|z)q(z)dz.

If the p(x,t|z) is generated by the vector field b(x,t|z) from p(x,0|z), then p(x,t) can be generated by b(x,t) defined as follows:

$$b\left(x,t\right):=E_{q}\left(z\right)\frac{b(x,t|z)p(x,0|z)}{p(x,t)}.$$

This is also intractable since p(x,t) is difficult to compute. The key is to introduce the conditional flow matching objective:

$$L_{\mathrm{CFM}}\left(\theta \right)=E_{t∼U(0,1),q(z),p(x,t|z)}{∥b_{\theta }\left(x,t\right)-b\left(x,t|z\right)∥}_{2}^{2}.$$

One can prove that $\nabla_{\theta }L_{\mathrm{CFM}}=\nabla_{\theta }L_{\mathrm{FM}}$, so training with CFM is equivalent with FM. The CFM objective is very useful when the b(x,t) is intractable but the conditional b(x,t|z) is tractable. So to approximate the dynamical optimal transport (11) is to use CFM. Assume $q\left(z\right)=q\left(z_{0},z_{1}\right)$, and set q(z) to be the Wasserstein optimal transport map π between the source distribution $\nu_{0}$ and the target distribution $\nu_{1}$, i.e., $q\left(z\right)=\pi \left(z_{0},z_{1}\right)$, where $z_{0}∼\nu_{0}$, $z_{1}∼\nu_{1}$. Then one can construct the Gaussian flow between $z_{0}$ and $z_{1}$ with standard deviation σ,

$$p\left(x,t|z\right)=N(x|tz_{1}+\left(1-t\right)z_{0}\left|\sigma^{2}\right),b\left(x,t|z\right)=z_{1}-z_{0}.$$

It can be proved that when σ→0, this also gives a way to solve the dynamical optimal transport [38]. The advantage of CFM is that it is simulation-free and can handle the thousand gene dimensions without reducing dimensionality.

**Schrödinger Bridge Problem** **(**g=0,σ≠0**)** In this case, the model can account for the stochastic effects, yet without unbalanced effects. We employ the Schrödinger Bridge problem to model the SDE dynamics, i.e., the cellular dynamics are $dx_{t}=b\left(x_{t},t\right)dt+\sigma \left(x_{t},t\right)dw_{t}$. The Schrödinger Bridge problem seeks to determine the most probable evolution between a specified initial distribution $\nu_{0}$ and a terminal distribution $\nu_{1}$ (assumed to possess a density in this study) relative to a given reference stochastic process. Formally, this problem is formulated as the minimization of the Kullback–Leibler (KL) divergence from the perspective of optimal control [142], as shown below:

(12) $$\min_{\mu_{0}^{X}=\nu_{0},\mu_{1}^{X}=\nu_{1}}D_{\mathrm{KL}}\left(\mu_{\left[0,1\right]}^{X}|\mu_{\left[0,1\right]}^{Y}\right),$$

where $\mu_{\left[0,1\right]}^{X}$ denotes the probability measure induced by the stochastic process $x_{t}$ for 0≤t≤1, defined on the space of all continuous paths $C(\left[0,1\right],R^{d})$. The distribution of $x_{t}$ at a given time t is characterized by the measure $\mu_{t}^{X}$ with density function p(x,t). The reference measure $\mu_{\left[0,1\right]}^{Y}$ is chosen as the probability measure induced by the process $dY_{t}=\sigma \left(Y_{t},t\right)dw_{t},$ where $w_{t}\in R^{d}$ represents the standard multidimensional Brownian motion.

Interestingly, the problem can be equivalently transformed into a dynamical form [39,40,142,143]

(13) $$\underset{\left(p,b\right)}{\inf}\int_{0}^{1}\int_{R^{d}}\left[\frac{1}{2}b^{⊤}\left(x,t\right)a{\left(x,t\right)}^{-1}b\left(x,t\right)\right]p\left(x,t\right)dxdt,$$

where the infimum is taken over all pairs of functions (p,b) satisfying $p\left(\cdot ,0\right)=\nu_{0}$, $p\left(\cdot ,1\right)=\nu_{1}$, and p(x,t) is absolutely continuous with respect to time. Additionally, the pair (p,b) must satisfy the Fokker–Planck Equation (3). We denote minimization problem (13) and the constraints (3) as the *dynamic diffusion Schrödinger bridge* formulation. Methods for modeling stochastic dynamics based on it have been widely developed [84,88,91,123,134,136], involving neural SDE, neural ODE, or flow matching techniques. We will next provide an overview of the methodologies in these approaches.

##### Neural SDE Solver

Similar to dynamical OT, one can solve the dynamical SB problem through the CNF formulation

$$\underset{\left(p(x,t),b(x,t)\right)}{\inf}E_{x_{0}∼\nu_{0}}\int_{0}^{1}\left[\frac{1}{2}b^{⊤}\left(x\left(t\right),t\right)a{\left(x\left(t\right),t\right)}^{-1}b\left(x\left(t\right),t\right)\right]dt+\lambda D\left(p\left(x,1\right),\nu_{1}\right).$$

Building upon this, one can parametrize b(x,t) and σ(x,t) using neural networks respectively and solve this formulation through POT and the neural SDE solver. However, besides these two terms, some work also introduces the idea of the principle of least action along the trajectory in which the optimal path has the smallest action value [84,123]. Thus, they introduce a new Hamilton–Jacobi–Bellman (HJB) regularization term [84] when assuming $b\left(x,t\right)=-\nabla_{x}\Phi \left(x,t\right)$, i.e.,

$$R_{h}=\int_{0}^{1}\int_{R^{d}}\left|\partial_{t}\Phi \left(x,t\right)-{∥\nabla_{x}\Phi \left(x,t\right)∥}_{2}^{2}\right|p\left(x,t\right)dxdt.$$

or a general form derived in [123].

##### Shrödinger Bridge Conditional Flow Matching

By leveraging CFM techniques, the simulation-free Shrödinger bridge [136] has also been recently developed. The core idea is to decompose the problem into a sequence of elementary conditional subproblems, each of which is more tractable, and subsequently express the overall solution as a mixture of the solutions to these conditional subproblems. Let the reference process be a Brownian motion (i.e., Y=σW). In this case, the Schrödinger bridge problem admits a unique solution $P^{*}$, which is expressed as a mixture of Brownian bridges weighted by an entropic optimal transport (OT) plan:

(14) $$P^{*}\left({\left(x_{t}\right)}_{t\in [0,1]}\right)=\int W\left(\left(x_{t}\right)|x_{0},x_{1}\right)\;d\pi_{2\sigma^{2}}^{★}\left(x_{0},x_{1}\right),$$

where $W\left(\left(x_{t}\right)|t\in (0,1)|x_{0},x_{1}\right)$ denotes the Brownian bridge between $x_{0}$ and $x_{1}$ with a diffusion rate σ, and $\pi_{2\sigma^{2}}^{★}\left(x_{0},x_{1}\right)$ represents the entropic optimal transport plan between the distributions. The calculation of $W\left(\left(x_{t}\right)|t\in (0,1)|x_{0},x_{1}\right)$ can be framed as an optimal control problem:

$$\begin{array}{cc} \; & \min_{b}E\int_{0}^{1}{∥b\left(x_{t},t\right)∥}^{2}\;dt,\\ \; & dx_{t}=b\left(x_{t},t\right)dt+\sigma dw_{t},\\ \; & X_{0}∼\delta_{x_{0}},\;X_{1}∼\delta_{x_{1}}, \end{array}$$

where $\delta_{x_{0}}$ and $\delta_{x_{1}}$ are Dirac delta functions centered at $x_{0}$ and $x_{1}$, respectively.

Assume σ is constant and then the corresponding Fokker–Planck equation in (12) yields

$$\partial_{t}p\left(x,t\right)=-\nabla_{x}\cdot \left(p(x,t)b(x,t)\right)+\frac{1}{2}\sigma^{2}\Delta p\left(x,t\right).$$

From this equation, it can be derived that the ODE

(15) $$dX_{t}=\underset{v(X_{t},t)}{\underset{︸}{\left(b\left(X_{t},t\right)-\frac{1}{2}\sigma^{2}\nabla_{x}\log p\left(X_{t},t\right)\right)}}dt,$$

together with the initial distribution generate the same distribution as SDE. The (15) is called the probability flow ODE. Conversely, if the probability flow ODE v(x,t) and $\nabla_{x}\log p\left(x,t\right)$ (also known as score function) are known, one can recover the SDE drift through $v\left(x,t\right)=b\left(x,t\right)+\frac{1}{2}\sigma^{2}\nabla_{x}\log p\left(x,t\right)$. So the flow-matching objective is

$$L_{U{\left[\mathrm{SF}\right]}^{2}M}\left(\theta \right)=E\left[\underset{\mathrm{flow}\;\mathrm{matching}\;\mathrm{loss}\;}{\underset{︸}{{∥v_{\theta }\left(x,t\right)-v\left(x,t\right)∥}^{2}}}+\lambda {\left(t\right)}^{2}\underset{\mathrm{score}\;\mathrm{matching}\;\mathrm{loss}\;}{\underset{︸}{{∥\nabla s_{\theta }\left(x,t\right)-\nabla \log p\left(x,t\right)∥}^{2}}}\right].$$

However, this loss is intractable; by (14) and the CFM objective, one can transform it into a tractable loss

$$L_{{\left[\mathrm{SF}\right]}^{2}M}\left(\theta \right)=E_{Q^{\prime }}\underset{\mathrm{conditional}\;\mathrm{flow}\;\mathrm{matching}\;\mathrm{loss}\;}{\underset{︸}{{∥v_{\theta }\left(x,t\right)-v(x,t|\left(x_{0},x_{1}\right)∥}^{2}}}+E_{Q^{\prime }}\lambda {\left(t\right)}^{2}\underset{\mathrm{conditional}\;\mathrm{score}\;\mathrm{matching}\;\mathrm{loss}\;}{\underset{︸}{{∥\nabla s_{\theta }\left(x,t\right)-\nabla \log p(x,t|\left(x_{0},x_{1}\right)∥}^{2}}},$$

where $Q^{\prime }=t∼U\left(0,1\right)⊗q\left(x_{0},x_{1}\right)⊗p\left(x,t|\left(x_{0},x_{1}\right)\right)$. Since the conditional path is a Brownian bridge, the analytic form can be derived, i.e., $p\left(x,t|\left(x_{0},x_{1}\right)\right)=N\left(x;tx_{1}+\left(1-t\right)x_{0},\sigma^{2}t\left(1-t\right)\right)$ and

$$v\left(x,t|\left(x_{0},x_{1}\right)\right)=\frac{1-2t}{t(1-t)}\left(x-\left(tx_{1}+\left(1-t\right)x_{0}\right)\right)+\left(x_{1}-x_{0}\right),$$

$$\nabla_{x}\log p\left(x,t|\left(x_{0},x_{1}\right)\right)=\frac{tx_{1}+\left(1-t\right)x_{0}-x}{\sigma^{2}t\left(1-t\right)},\;t\in \left[0,1\right].$$

And $q(x_{0},x_{1})$ can be computed by the entropic optimal transport.

**Unbalanced Wasserstein–Fisher–Rao metric**  **(** g≠0,σ=0**)** In this case, the model can account for the unbalanced dynamics, however, it can not account for stochastic dynamics. The cellular dynamics are also governed by the ODE model by $dx_{t}=b\left(x_{t},t\right)dt$. Then, one can use the dynamical unbalanced optimal transport to model these dynamics, also known as Wasserstein–Fisher–Rao metric [144,145,146], which can be stated as

(16) $$\begin{array}{c} \underset{\left(p(x,t),b(x,t),g(x,t)\right)}{\inf}\int_{0}^{1}\int_{R^{d}}\left(\frac{1}{2}{∥b\left(x,t\right)∥}_{2}^{2}+\alpha {\left|g\left(x,t\right)\right|}_{2}^{2}\right)p\left(x,t\right)dxdt,\\ s.t.\partial_{t}p+\nabla \cdot \left(b(x,t)p\right)=g\left(x,t\right)p,\;p{{|}_{t=0}=\nu_{0},p|}_{t=1}=\nu_{1}. \end{array}$$

Here, α denotes a hyperparameter that controls the weighting. It is also important to note that in this context, $\nu_{0}$ and $\nu_{1}$ do not necessarily correspond to normalized probability densities; rather, they generally represent mass densities.

Recent works such as TrajectoryNet and TIGON utilize (16) to infer unbalanced dynamics from scRNA-seq data [81,86]. To derive a CNF solver for (16), TIGON [81] observes that along the characteristic line $\frac{dx}{dt}=b\left(x,t\right)$, one has

$$\int_{0}^{1}\int_{R^{d}}f\left(x,t\right)p\left(x,t\right)dxdt=E_{x_{0}∼p_{0}}\int_{0}^{1}f\left(x,t\right)e^{\int_{0}^{t}g\left(x,s\right)ds}dt$$

and $\frac{d(\ln p)}{dt}=g-\nabla \cdot v$. This can make the computation of both energy loss and reconstruction loss in high dimensional space tractable. Therefore, one can parameterize b(x,t) and g(x,t) using neural networks, respectively, and train them by minimizing the overall loss.

**Regularized Unbalanced Optimal Transport** **(**g≠0,σ≠0**)** In this case, the model can account for both the unbalanced and stochastic dynamics. The cellular dynamics are governed by the SDE model $dx_{t}=b\left(x_{t},t\right)dt+\sigma \left(t\right)Idw_{t}$. We can use the regularized unbalanced optimal transport to model it [40,138]. It can be viewed as an unbalanced relaxation of the dynamic formulation of the Schrödinger bridge problem. Consider

(17) $$\underset{\left(p,b,g\right)}{\inf}\int_{0}^{1}\int_{R^{d}}\frac{1}{2}{∥b(x,t)∥}_{2}^{2}p\left(x,t\right)dxdt+\int_{0}^{1}\int_{R^{d}}\alpha \Psi \left(g(x,t)\right)p\left(x,t\right)dxdt,$$

where Ψ:R→[0,+∞] corresponds to the growth penalty function, the infimum is taken over all pairs $\left(p,b\right)$ such that p(·,0)=$\nu_{0},p\left(\cdot ,1\right)=\nu_{1},p\left(x,t\right)$ absolutely continuous, and

(18) $$\partial_{t}p=-\nabla_{x}\cdot \left(pb\right)+\frac{1}{2}\nabla_{x}^{2}:\left(\sigma^{2}\left(t\right)Ip\right)+gp$$

with vanishing boundary condition: $\lim_{|x|\to \infty }p\left(x,t\right)=0$.

One can similarly develop a dynamical OT solver relying on a neural SDE solver, which might be less efficient compared to a neural ODE solver. Recently, DeepRUOT [40] reformulates the RUOT problem with the Fisher information regularization, equivalently expressed as

(19) $$\underset{\left(p,v,g\right)}{\inf}\int_{0}^{1}\int_{R^{d}}\left[\frac{1}{2}{∥v(x,t)∥}_{2}^{2}+\frac{\sigma^{4}\left(t\right)}{8}{∥\nabla_{x}\log p∥}_{2}^{2}-\frac{\sigma^{2}\left(t\right)}{2}\left(1+\log p\right)g+\alpha \Psi \left(g\right)\right]p\left(x,t\right)dxdt,$$

where the infimum is taken over all triplets $\left(p,v,g\right)$ such that p(·,0)=$\nu_{0},p\left(\cdot ,1\right)=\nu_{1},p\left(x,t\right)$ absolutely continuous, and

(20) $$\partial_{t}p=-\nabla_{x}\cdot \left(pv(x,t)\right)+g\left(x,t\right)p$$

with vanishing boundary condition: $\lim_{|x|\to \infty }p\left(x,t\right)=0$. Here v(x,t) is a new vector field, representing the probability flow ODE field.

Thus, the original SDE $dx_{t}=\left(b\left(x_{t},t\right)\right)dt+\sigma \left(t\right)dw_{t}$ now can be transformed into the probability flow ODE

$$dx_{t}=\underset{v\left(x_{t},t\right)}{\underset{︸}{\left(b\left(x_{t},t\right)-\frac{1}{2}\sigma^{2}\left(t\right)\nabla_{x}\log p\left(x_{t},t\right)\right)}}dt.$$

If the probability flow ODE’s drift v(x,t), σ(t) and the *score function* $\nabla_{x}\log p\left(x,t\right)$ are specified, then the the drift term b(x,t) of the SDE can be recovered by $b\left(x,t\right)=v\left(x,t\right)+\frac{1}{2}\sigma^{2}\left(t\right)\nabla_{x}\log p\left(x,t\right).$ Therefore, specifying an SDE is equivalent to specifying the probability flow ODE and the score function $\nabla_{x}\log p\left(x,t\right)$. One can then use neural networks $v_{\theta },\;g_{\theta }$ and $s_{\theta }$ to parameterize v(x,t), g(x,t), and $\frac{1}{2}\sigma^{2}\left(t\right)\log p\left(x,t\right)$, respectively.

To train DeepRUOT, the overall loss is composed of three parts, i.e., the energy loss, reconstruction loss, and the Fokker–Planck constraint:

(21) $$L=L_{\mathrm{Energy}}+\lambda_{r}L_{\mathrm{Recons}}+\lambda_{f}L_{\mathrm{FP}}.$$

The $L_{\mathrm{Energy}}$ loss aims for the least action of kinetic energy in Equation (19), which can be computed via CNF by adopting the similar approach in TIGON [81]. The reconstruction loss $L_{\mathrm{Recons}}$ aims the dynamics to match data distribution at the later time point (i.e., $p\left(\cdot ,1\right)=\nu_{1}$). To achieve the matching in unbalanced settings, DeepRUOT further decomposes it into two parts:

(22) $$L_{\mathrm{Recons}}=\lambda_{m}L_{\mathrm{Mass}}+\lambda_{d}L_{\mathrm{OT}}$$

where $L_{\mathrm{Mass}}$ aims to align the number of cells and $L_{\mathrm{OT}}$ uses normalized weights to perform optimal transport matching. Lastly, $L_{\mathrm{FP}}$ aims to let the three parameterized neural networks satisfy the Fokker–Planck constraints (20). DeepRUOT first utilizes a Gaussian mixture model to estimate the initial distribution, ensuring that it satisfies the initial conditions $p_{0}$, and the physics-informed (PINN) loss [147] is defined as

(23) $$L_{\mathrm{FP}}=∥\partial_{t}p_{\theta }+\nabla_{x}\cdot \left(p_{\theta }v_{\theta }\right)-g_{\theta }p_{\theta }∥+\lambda_{w}∥p_{\theta }\left(x,0\right)-p_{0}∥,p_{\theta }=\exp\frac{2}{\sigma^{2}}s_{\theta }.$$

In [40], DeepRUOT adopts a two-stage training approach to stabilize the training process. For the pre-training stage, they use reconstruction loss only to train $v_{\theta }$ and $g_{\theta }$. Then, they fix $v_{\theta }$ and $g_{\theta }$ and employ conditional flow-matching [37,38,136] to learn the log density function ($s_{\theta }\left(x,t\right)$). Finally, for the training stage, they use the $v_{\theta }$, $g_{\theta }$, and the log density function as the starting point, then obtain the final result by minimizing the total loss (21).

## 4. Dynamic Modeling of Spatial Transcriptomics

**Figure 4:**
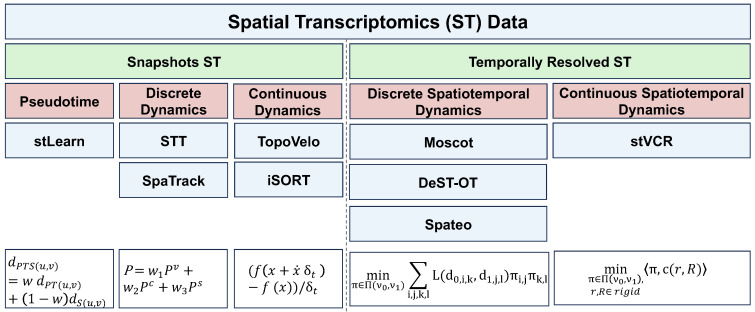
**Dynamic modeling of spatial transcriptomics**.

In this section, we review the dynamical modeling approaches for spatial transcriptomics data. We will first present several random walk- or ODE-based methods to model the snapshot spatial data. Next, we focus on the recent progress to dissect the spatiotemporal dynamics underlying datasets with both space and time resolutions. Figure 4 provides an overview of these spatial transcriptomics modeling approaches.

### 4.1. Snapshot Spatial Transcriptomics

Below we describe several modeling strategies for single snapshot spatial transcriptomics data, including pseudotime, random walk, and continuous differential equation models, respectively.

#### 4.1.1. Pseudotime Methods

In the context of spatiotemporal trajectory inference, stLearn [148] proposes the PSTS algorithm that combines spatial information and geodesic-based pseudotime information to infer the spatiotemporal developmental trajectory of cells. The pseudotime distance between two clusters is defined as

$$d_{\mathrm{PT}}\left(u,v\right)=\frac{1}{n}\sum_{i=1}^{n}\sum_{j=1}^{n}\left(1-\frac{p_{u,i}\cdot p_{v,i}}{∥p_{u,i}∥\cdot ∥p_{v,j}∥}\right),$$

where $p_{u,i}$ and $p_{v,i}$ are the PCA vectors of gene expression data points in two clusters. The spatial distance is defined as the Euclidean distance between the centroids of the two clusters. The spatiotemporal distance between clusters is the weighted sum of pseudotime distance and spatial distance

$$d_{\mathrm{PTS}}\left(u,v\right)=\omega d_{\mathrm{PT}}\left(u,v\right)+\left(1-\omega \right)d_{S}\left(u,v\right).$$

Each cluster is then treated as a node in a graph, and the edge weights are determined by $d_{\mathrm{PTS}}$. By optimizing edge selection using a minimum spanning tree, the optimal trajectory structure can be identified.

#### 4.1.2. Discrete Spatial Dynamics Modeling

STT [149] is a random walk-based algorithm to detect multi-stable attractors in spatial transcriptomics. Central to STT is the incorporation of a space coordinate-aware random walk, with the transition probability matrix having the form $P=w_{1}P_{v}+w_{2}P_{c}+\left(1-w_{1}-w_{2}\right)P_{s}$, where $P_{v}$ is induced by an attractor-specific RNA velocity (named spatial transition tensor), $P_{c}$ is induced by gene expression similarity (i.e., diffusion in the gene space), and $P_{s}$ is induced by space coordinates (i.e., diffusion in the physical space). By iteratively (1) decomposing *P* to identify attractors and assign attractor membership to each individual cell and (2) improving attractor-specific RNA velocity estimation, STT is able to identify transitional cells in the snapshot spatial transcriptomics data and plot the local streamlines within attractors.

SpaTrack [150] is a spatial transcriptomics analysis tool based on optimal transport theory, which reconstructs cell differentiation trajectories by integrating gene expression profiles and spatial coordinates of cells. When processing Snapshot data, SpaTrack defines the transition cost matrix between cells by weighting gene expression distance and spatial distance as follows: $C_{ij}=\alpha_{1}∥g_{i}-g_{j}{∥}^{2}+\alpha_{2}{∥z_{i}-z_{j}∥}^{2}$ where $g_{i}$ represents the gene expression of cell *i*, $x_{i}$ denotes the spatial coordinates of cell *i*, and $\alpha_{1},\alpha_{2}$ are weighting coefficients. The transition probability matrix between cells is obtained by solving the following entropy-regularized optimal transport (OT) problem

$$P=\arg\min_{P}\sum_{ij}C_{ij}P_{ij}+\epsilon H\left(P\right)\;s.t.\sum_{i}P_{ij}=1,\sum_{j}P_{ij}=1$$

where H(P) denotes the entropy regularization term and ϵ is the regularization coefficient. SpaTrack identifies trajectory starting points using single-cell entropy. Let the identified starting points be cells 1,2,⋯,s. The probability of transitioning from starting cells to cell *i* can be calculated as $\gamma_{i}=\sum_{j=1}^{s}P_{ji}$. By sorting cells in ascending order based on their $\gamma_{i}$ values, the position of each cell in the differentiation trajectory can be determined.

#### 4.1.3. Continuous Spatial Dynamics Modeling

Several methods aim to extend the continuous RNA velocity model of scRNA-seq data toward snapshot spatial transcriptomics. One recent method, iSORT [151] uses transfer learning to obtain a mapping of gene expression to spatial location z=f(x) and proposes the concept of spatial RNA velocity, which utilizes the velocity of gene expression and the mapping z=f(x) to obtain spatial RNA velocity, formally

$$\frac{dz}{dt}=\nabla f\cdot \frac{dx}{dt}.$$

In addition, Topovelo [152] uses a graph neural network to infer RNA velocity for spatial transcriptomics data and suggests that a decoder could be trained to further infer continuous spatial velocities.

### 4.2. Temporally Resolved Spatial Transcriptomics

The availability of time-series ST data opens new avenues to explore cellular migration within physical space [41,152,153]. Nevertheless, the inherently destructive nature of sequencing limits ST data to static snapshots rather than continuous trajectories. Particularly, when sequencing is performed at various time points during embryonic development, the resulting time-series ST data are often derived from distinct biological samples, leading to multiple unpaired snapshots [17,154,155]. In addition, due to possible rotation, translation, and stretching of different slices, the spatial coordinates of different samples are not in the same coordinate system [41,153,156]. Therefore, reconstructing trajectories of cell state transition, proliferation, and migration for time-series ST data is a challenging task.

To overcome these challenges, many methods have been developed in recent years. Similar to modeling temporal single-cell data, these methods can be divided into two categories: those that model dynamics on discrete cell states [36,153,157] and those that model dynamics in continuous spaces [41].

#### 4.2.1. Discrete Spatiotemporal Dynamics Modeling

Among the methods that model spatiotemporal dynamics on discrete cell states, recent work includes Moscot [36], DeST-OT [157], and Spateo [153]. These approaches employ fused Gromov–Wasserstein optimal transport [158,159] as the main tool, which was first used by PASTE [156] to align adjacent 2D slices to reconstruct the 3D structure of the tissue.

**Fused Gromov–Wasserstein Optimal Transport** We consider two adjacent unpaired slices (X,Z) and $\left(X^{\prime },Z^{\prime }\right)$ with spots (or cell) numbers *N* and *M*, where $X\in R^{N\times G}$, $X^{\prime }\in R^{M\times G}$ are the gene expression of the two slices, and $Z\in R^{N\times 2}$ and $Z^{\prime }\in R^{M\times 2}$ are the spatial coordinates. In addition, the spatial coordinates of each slice can be converted into a distance matrix $D\in R_{+}^{N\times N}$, where $d_{ij}={∥z_{i}-z_{j}∥}_{2}$. The fused Gromov–Wasserstein optimal transport problem reads as follows:

(24) $$\begin{array}{c} \min_{\pi \in \Pi (\nu_{0},\nu_{1})}\left(1-\alpha \right)\sum_{i,j}c_{i,j}\pi_{i,j}+\alpha \sum_{i,j,k,l}{\left(d_{i,k}-d_{j,l}^{\prime }\right)}^{2}\pi_{i,j}\pi_{k,l}, \end{array}$$

where $\pi ,\Pi (\nu_{0},\nu_{1})$ and *c* have the same meaning as before, and α is a hyperparameter that weighs the importance of gene expression and spatial location. The fused Gromov–Wasserstein optimal transport (FGW-OT) problems can also be solved by calling the POT (version 0.9.5) package [92].

**Generalized Weighted Procrustes Problem** When the mapping π is found, in order to unify the spatial coordinates of adjacent slices into the same coordinate system by a rigid body transformation (rotation and translation), we need to solve a generalized weighted Procrustes problem. Formally,

(25) $$\hat{R},\hat{r}=\underset{\begin{array}{c} R\in R^{2\times 2},r\in R^{2}\\ R^{T}R=I,\det R=1 \end{array}}{\arg\min}\sum_{i,j}\pi_{ij}{∥z_{i}-\left(Rz_{j}^{\prime }+r\right)∥}_{2}^{2},$$

where R is the rotation matrix and r is the translation vector.

**Applications in ST Data** Moscot [36] extends FGW-OT to model slices of adjacent time points by adding the entropy regularization mentioned in Equation (8) and the unbalanced settings mentioned in Equations (9) and (10). Formally,

$$\begin{array}{cc} \min_{\pi \in R_{+}^{N\times M}} & \left(1-\alpha \right)\sum_{i,j}c_{i,j}\pi_{i,j}+\alpha \sum_{i,j,k,l}{\left(d_{i,k}-d_{j,l}^{\prime }\right)}^{2}\pi_{i,j}\pi_{k,l}\\ \; & +\tau_{1}\mathrm{KL}(\pi 1_{M}\left|\right|\nu_{0})+\tau_{2}\mathrm{KL}(\pi^{T}1_{N}\left|\right|\nu_{1})-\varepsilon H\left(\pi \right), \end{array}$$

where $\tau_{1},\tau_{2},\varepsilon$ and H(π) have the same meaning as before and $\nu_{0}$ is calculated from a pre-selected gene set according to Equation (10). Note that when we talk about ST data at different time points, the spatial coordinates can not only be 2D but can also be reconstructed in 3D. We use $d_{\mathrm{spa}}$ to refer to the dimension of the spatial coordinates.

DeST-OT [157] designs methods that enable simultaneous inference of cell growth rate and mapping from data. The DeST-OT optimization problem with the semi-relaxed constraints and entropic regularization is

$$\begin{array}{cc} \min & E_{\mathrm{DeST}-\mathrm{OT}\;}\left(\pi \right)+\tau_{1}\mathrm{KL}(\pi 1_{M}\left|\right|\nu_{0})-\varepsilon H\left(\pi \right)\\ \;s.t.\; & \pi^{T}1_{N}=\nu_{1},\;\pi \in R_{+}^{N\times M}, \end{array}$$

where $E_{\mathrm{DeST}-\mathrm{OT}\;}\left(\pi \right)$ includes the Wasserstein OT term and another term $E^{M}\left(\pi \right)$ related to growth and GW-OT, that is,

$$E_{\mathrm{DeST}-\mathrm{OT}\;}:=\left(1-\alpha \right)\sum_{i,j^{\prime }}c_{i,j^{\prime }}\pi_{i,j^{\prime }}+\alpha E^{M}\left(\pi \right).$$

$E^{M}\left(\pi \right)$ is defined as

$$\begin{array}{cc} E^{M}\left(\pi \right):=\frac{1}{2} & \left(\sum_{i,j^{\prime },k^{\prime }}\pi_{ij^{\prime }}\pi_{ik^{\prime }}M_{j^{\prime }k^{\prime }}^{\prime 2}+\sum_{i,j,k^{\prime }}\pi_{ik^{\prime }}\pi_{jk^{\prime }}M_{ij}^{2}\right.\\ \; & \left.+\sum_{i,j^{\prime },k,l^{\prime }}{\left(M_{ik}-M_{j^{\prime }l^{\prime }}^{\prime }\right)}^{2}\pi_{ij^{\prime }}\pi_{kl^{\prime }}\right), \end{array}$$

where $M=D^{\mathrm{spa}}⊙D^{\exp}$ measures the distance between two cells in the same slice, and $D^{\mathrm{spa}}$ and $D^{\exp}$ are distance matrices constructed on each slice according to spatial coordinates S and gene expression X, respectively. That is, $d_{i,j}^{\mathrm{spa}}={∥z_{i}-z_{j}∥}_{2}$ and $d_{i,j}^{\exp}={∥x_{i}-x_{j}∥}_{2}$. In addition, the first and second terms in $E^{M}\left(\pi \right)$ promote the proximity of different descendants of a cell at the previous moment and the proximity of different ancestors of a cell at the later moment, respectively, and the third term is the usual GW term that only replaces the spatial distance matrix D with the M distance matrix. In DeST-OT, the authors define the growth vector $\xi =\pi 1_{M}-\nu_{0}$ and the growth rate $g=\log\left(1+N\xi \right)/\left(t_{1}-t_{0}\right)$.

Spateo [153] uses maps π obtained by other OT-based methods to unify spatial coordinates at different times into the reference coordinate system by solving the generalized weighted Procrustes problem in Equation (25) (possibly in 3D). Next, Spateo selects the spatial coordinates of the cell with the most weight at the late time point mapped from each cell at the early time point as its future state, formally

$$z_{i,\mathrm{future}}=z_{\arg\max\pi_{i,:}}^{\prime },$$

where $\pi_{i,:}$ refers to the *i* row of π, that is, the weight of the *i* cell mapped from the early time point to each cell at the late time point. When the future spatial coordinates of each cell are determined, we can define the spatial velocity of each cell

$$v_{i}^{\mathrm{spa}}=z_{i,\mathrm{future}}-z_{i}.$$

Finally, Spateo recovers a continuous spatial velocity field $\frac{dz}{dt}=f\left(z\right),$ from the spatial velocity of each cell, allowing for a series of differential geometry analyses, including divergence, acceleration, curvature, and torsion.

#### 4.2.2. Spatiotemporal Dynamics Modeling

The majority of current approaches model spatial coordinates based on Gromov–Wasserstein OT, which has no dynamic form. Recently, stVCR [41] proposes to model spatial coordinates using rigid-body transformation invariant OT, as well as using the widely used Wasserstein OT for modeling gene expression and unbalanced OT for modeling cellular proliferation. Next, stVCR integrates all modules into dynamic forms, making it possible to reconstruct dynamic continuous trajectories of cell differentiation, migration, and proliferation simultaneously.

*Rigid body transformation invariant optimal transport* The method in [160] considers the optimal transport problem invariant to a given set of manipulations G. It simultaneously searches for the optimal mapping π and the optimal transformation *g* through the optimization problem:

(26) $$\left(\pi^{★},g^{★}\right)=\underset{\pi \in \Pi (\nu_{0},\nu_{1}),g\in G}{\arg\min}\langle c\left(g\right),\pi \rangle \overset{\;\mathrm{def}.\;}{=}\sum_{i,j}\pi_{i,j}d\left(z_{i},g\left(Z_{j}^{\prime }\right)\right).$$

Solving problem (26) directly is difficult, and it can be solved iteratively by

(27) $$\begin{array}{cc} \pi^{\left(n\right)} & =\underset{\pi \in \Pi (\nu_{0},\nu_{1})}{\arg\min}\sum_{i,j}\pi_{ij}d\left(z_{i},g^{\left(n\right)}\left(Z_{j}^{\prime }\right)\right), \end{array}$$

(28) $$\begin{array}{cc} g^{\left(n+1\right)} & =\underset{g\in G}{\arg\min}\sum_{i,j}\pi_{ij}^{\left(n\right)}d\left(z_{i},g\left(Z_{j}^{\prime }\right)\right). \end{array}$$

The subproblem (27) is to solve a static OT. In addition, when we choose the set G as the set of rigid body transformations, we call this problem rigid body transformation invariant optimal transport. At this point, subproblem (28) is the generalized weighted Procrustes problem mentioned before.

Consider the ST data $\left(Z^{\left(0:K\right)},X^{\left(0:K\right)}\right)$ at $t_{0},t_{1}\cdots t_{K}$ totaling *K* time points, and the number of cells in each observation is $n_{0},n_{1}\ldots n_{K}$. stVCR uses the spatial coordinate system of the data at $t_{0}$ as a reference, and searches for the optimal dynamics and the optimal rigid-body transformation $\left(r_{1:k},R_{1:k}\right)$ by interpolating the empirical probability distributions of the data after the rigid-body transformation ${\hat{p}}^{k}$ and the number of cells $n_{k}$ using a transport-with-growth partial differential equation (PDE)

(29) $$\partial_{t}p_{t}\left(z,x\right)+\nabla \cdot \left(\left(v_{t}\left(z,x\right),b_{t}\left(z,x\right)\right)p_{t}\left(z,x\right)\right)=g_{t}\left(z,x\right)p_{t}\left(z,x\right),$$

where $v_{t}\left(z,x\right)$ is cell spatial migration velocity, $b_{t}\left(z,x\right)$ is RNA velocity and $g_{t}\left(z,x\right)$ is cell growth rate. Thus, the feasible state space S for the arguments under constraints is

(30) $$\begin{array}{cc} S( & Z^{\left(0:K\right)},X^{\left(0:K\right)}):=\{\left(p_{t},v_{t},b_{t},g_{t};R_{1:K},r_{1:K}\right)|\;\partial_{t}p_{t}+\nabla \cdot \left(\left(v_{t},b_{t}\right)p_{t}\right)=g_{t}p_{t},\\ \; & p_{t_{0}}=p^{\left(0\right)},{∥p_{t_{k}}∥}_{1}=n_{k}/n_{0},{\bar{p}}_{t_{k}}=p^{\left(k\right)},R_{k}^{T}R_{k}=I,\det R_{k}=1,\;k=1,2,\ldots ,K\}, \end{array}$$

where $∥p_{t}{∥}_{1}:=\int p_{t}dzdx$ is the total mass of $p_{t}$ and ${\bar{p}}_{t}:=p_{t}/{∥p_{t}∥}_{1}$. stVCR finds optimal dynamics $\left(p_{t},v_{t},b_{t},g_{t}\right)$ and optimal rigid-body transformations $\left(R_{1:K},r_{1:K}\right)$ by minimizing the Wasserstein–Fisher–Rao (WFR) distance

(31) $$\int_{t_{0}}^{t_{K}}\int_{R^{G+d_{\mathrm{spa}}}}\left({∥v_{t}∥}^{2}+\alpha_{\mathrm{Exp}}{∥b_{t}∥}^{2}+\alpha_{\mathrm{Gro}}g_{t}^{2}\right)p_{t}\left(z,x\right)dzdxdt$$

for $\left(p_{t},v_{t},b_{t},g_{t};R_{1:K},r_{1:K}\right)\in S\left(Z^{\left(0:K\right)},X^{\left(0:K\right)}\right)$. According to the direct derivation of the solution of the Feynman–Kac type PDE (29) by characteristics, Equation (31) has a dimensionally independent form

(32) $$\begin{array}{cc} L_{\mathrm{Dyn}}=E_{\left(z^{\left(t_{0}\right)},x^{\left(t_{0}\right)}\right)∼p^{\left(0\right)}}\int_{t_{0}}^{t_{K}}( & ∥v_{t}\left(z^{\left(t\right)},x^{\left(t\right)}\right){∥}^{2}+\alpha_{\mathrm{Exp}}{∥b_{t}\left(z^{\left(t\right)},x^{\left(t\right)}\right)∥}^{2}\\ \; & +\alpha_{\mathrm{Gro}}{∥g_{t}\left(z^{\left(t\right)},x^{\left(t\right)}\right)∥}^{2})w_{t}\left[z,x\right]dt, \end{array}$$

where $z^{\left(t\right)},x^{\left(t\right)},w_{t}\left[z,x\right]$ satisfies the characteristic ordinary differential equations (ODEs)

(33) $$\begin{array}{cc} \frac{dz^{\left(t\right)}}{dt}=v_{t}\left(z^{\left(t\right)},x^{\left(t\right)}\right),\;\frac{dx^{\left(t\right)}}{dt} & =b_{t}\left(z^{\left(t\right)},x^{\left(t\right)}\right),\;\frac{d\ln w_{t}}{dt}=g_{t}\left(z^{\left(t\right)},x^{\left(t\right)}\right),\\ \left(z^{\left(t\right)},x^{\left(t\right)},w_{t}\right){|}_{t=t_{0}} & =(z^{\left(t_{0}\right)},x^{\left(t_{0}\right)},1). \end{array}$$

stVCR implemented the constraints $∥p_{t_{k}}{∥}_{1}=n_{k}/n_{0}$ and ${\bar{p}}_{t_{k}}={\hat{p}}^{\left(k\right)}$ in Equation (30) as soft penalties by performing distribution matching

(34) $$L_{\mathrm{Mch}}=\sum_{k=1}^{K}{\left(W_{2}\left({\bar{p}}_{t_{k}},{\hat{p}}^{\left(k\right)}\right)\right)}^{2}+\kappa_{\mathrm{Gro}}\sum_{k=1}^{K}\frac{|\sum_{j=1}^{n_{0}}w_{t_{k},j}-n_{k}|}{n_{k}},$$

where the second term promotes a reduction in the relative error of the total mass, and the first term is the 2-Wasserstein distance between the normalized distribution corresponding to the dynamics ${\bar{p}}_{t_{k}}$ and the probability distribution of the observed data after rigid body transformation ${\hat{p}}^{\left(k\right)}$, where the cost function is defined as

$$c_{ij}^{\left(k\right)}=\kappa_{\mathrm{Exp}}∥x_{i}^{\left(t_{k}\right)}-{\hat{x}}_{j}^{\left(k\right)}{∥}_{2}^{2}+\left(1-\kappa_{\mathrm{Exp}}\right){∥z_{i}^{\left(t_{k}\right)}-{\hat{z}}_{j}^{\left(k\right)}∥}_{2}^{2},\;i=1:n_{0},j=1:n_{k},$$

where $\kappa_{\mathrm{Exp}}$ weighs the importance of gene expression and spatial coordinates in distribution matching. In addition, for annotated data, stVCR achieves modeling of known type transitions by modifying $L_{\mathrm{Mch}}$, and spatial structure preservation for specified organs or tissues by adding an optional objective function $L_{\mathrm{SSP}}^{\mathrm{opt}}$.

In summary, the loss function of stVCR contains two required items and one optional item

(35) $$L=L_{\mathrm{Dyn}}+\lambda_{\mathrm{Mch}}L_{\mathrm{Mch}}+\lambda_{\mathrm{SSP}}L_{\mathrm{SSP}}^{\mathrm{opt}}.$$

In practice, stVCR parameterizes the dynamics $v_{t}\left(z^{\left(t\right)},x^{\left(t\right)}\right)$, $b_{t}\left(z^{\left(t\right)},x^{\left(t\right)}\right)$ and $g_{t}\left(z^{\left(t\right)},x^{\left(t\right)}\right)$ into neural networks as well as parameterizes the rotation matrix $R_{1:K}$ into rotation angles $\alpha_{1:K}$ (or Euler angles $\left(\alpha_{1:K},\beta_{1:K},\gamma_{1:K}\right)$ in 3D) and solves iteratively using back-propagation algorithm.

## 5. Extensions, Challenges, and Future Directions

Recent advancements in single-cell transcriptomics, spatial transcriptomics, and computational modeling have significantly improved our ability to reconstruct cellular dynamics. However, several outstanding challenges remain, particularly in integrating different discrete and continuous models, handling the complexity of single-cell dynamics, and ensuring the biological interpretability of inferred dynamical systems. This section discusses key areas for further development, focusing on new methodologies that combine discrete and continuous modeling approaches, the construction of comprehensive dynamical frameworks, and the broader applications for modeling cellular fate decisions.

### 5.1. Bridging Discrete and Continuous Dynamics Modeling

One interesting topic to explore is building connections between discrete dynamic models (e.g., Markov chain) with continuous differential equations when dealing with scRNA-seq data. In CellRank [62,63], the output from continuous models could help to refine the random walk on the data point cloud by introducing various kernels. Let the spliced RNA counts of cells *i* and *j* be $s_{i}$ and $s_{j}$, respectively. The **Gaussian Kernel** is defined as

$$d_{g}\left(s_{i},s_{j}\right)=\exp\left(-\frac{∥s_{i}-s_{j}{∥}^{2}}{\sigma^{2}}\right),$$

Let the position vector from cell *i* to cell *j* be $\delta_{ij}=s_{j}-s_{i}$ and $v_{i}$ denotes the estimated RNA velocity of cell *i*. Then, the three velocity kernels can be introduced as

- Cosine Kernel: $v_{\cos}\left(s_{i},s_{j}\right)=g\left(\cos(\delta_{ij},v_{i})\right)$,
- Correlation Kernel: $v_{\mathrm{corr}}\left(s_{i},s_{j}\right)=g\left(\mathrm{corr}(\delta_{ij},v_{i})\right)$,
- Inner Product Kernel: $v_{\mathrm{ip}}\left(s_{i},s_{j}\right)=g\left(\delta_{ij}^{T}v_{i}\right)$.

Here, *g* is a bounded, positive, monotonic increasing function such as an exponential function. In CellRank [62], the actual transition kernel can combine these two parts either by weighted summation or multiplication. For example, the kernel used in the original RNA velocity method is

$$\mathrm{Ker}\left(s_{i},s_{j}\right)=\lambda v_{\cos}\left(s_{i},s_{j}\right)+\left(1-\lambda \right)d_{g}\left(s_{i},s_{j}\right),$$

where λ is a weighting coefficient. The Markov chain transition matrix is constructed as

$$p_{ij}=\frac{\mathrm{Ker}(s_{i},s_{j})}{\sum_{j}\mathrm{Ker}\left(s_{i},s_{j}\right)}.$$

CellRank2 [63] provides more flexible options for the transition kernel and incorporates prior knowledge. For example, if pseudotime is known in advance, it can be used to adjust the transition kernel

$${\mathrm{Ker}}_{\mathrm{adj}}\left(s_{i},s_{j}\right)=\mathrm{Ker}\left(s_{i},s_{j}\right)f\left(\Delta t_{ij}\right),$$

where

$$f\left(\Delta t\right)=\left\{\begin{array}{cc} \frac{2}{\sqrt{1+\exp(b\Delta t)}} & \Delta t<0,\\ 1 & \Delta t\geq 0. \end{array}\right.$$

Additionally, a unified transition kernel can be constructed for multi-time point data. For data at different time points, a transport map such as optimal transport (OT) can be used to define $\pi_{t_{j},t_{j+1}}$. By placing the same time point data on the diagonal of a global transition matrix and the transport map between different time points on the off-diagonal, a global transition matrix *T* can be obtained, enabling the construction of a Markov chain across different time points.

Another direction is to analyze the theoretical convergence of discrete dynamics as the number of data points tends to infinity. A well-known example is the study of the continuum limit of diffusion map random walk [161], stating that the random walk induced by the Gaussian kernel would converge to the dynamics of the Fokker–Planck equation. When considering growth, the directed random walk defined by PBA would converge to (3). Interestingly, ref. [61] also proves that the coarse-grained transition probabilities yield the continuum limit of transition rate among attractors (6), therefore validating the rationale of MuTrans.

Once such a theoretical connection is built, new theoretical insights could be drawn toward the algorithm design. For instance, ref. [44] systematically investigates the continuous limit of RNA-velocity-induced random walk kernels. For example, if the transition kernel is $\mathrm{Ker}\left(s_{i},s_{j}\right)=d_{g}\left(s_{i},s_{j}\right)\cdot v_{\cos}\left(s_{i},s_{j}\right),$ the corresponding ODE yields the desired streamlined equation that correctly reveals the vector field directionality $\frac{dx}{dt}=\frac{v}{∥v∥}.$ Meanwhile, for $\mathrm{Ker}\left(s_{i},s_{j}\right)=d_{g}\left(s_{i},s_{j}\right)\cdot v_{\mathrm{corr}}\left(s_{i},s_{j}\right)$, the corresponding ODE is $\frac{dx}{dt}=\frac{P_{1}v}{∥P_{1}v∥},$ where P is the projection operator defined as $P_{n}x=\left(I-\hat{n}⊗\hat{n}\right)\cdot x,\;\hat{n}=\frac{n}{∥n∥},\;1={\left(1,1,\cdots ,1\right)}^{T}.$ which indicates that the correlation kernel might alter both the direction and magnitude of RNA velocity in the continuous limit.

Some approaches also use discrete graphs to represent the geometric structure of data [162]. Graphdynamo [163] and Graphvelo [164] propose a method that leverages geometric structure to correct RNA velocity. They assume that the cell data points $x_{i}$ lie on a low-dimensional manifold embedded in a high-dimensional space (in classical mechanics, this is known as the “configuration manifold”) and that each cell’s RNA velocity vector lies in the tangent space $T_{x}M$ at the point $x_{i}$ on the manifold. Let $\delta_{ij}$ denote the displacement vector from cell *i* to its neighboring cell *j*. With a sufficient number of such $\delta_{ij}$, one can construct a non-orthogonal normalized basis for $T_{x}M$. Thus, the RNA velocity vector in $T_{x}M$ can be expressed as $v_{\|}\left(x_{i}\right)=\sum_{j\in N_{i}}\phi_{ij}\delta_{ij}$ The coefficients of the linear combination, $\phi_{i}=\left\{\phi_{ij}|j\in N_{i}\right\}$, are determined by minimizing the following loss:

$$L\left(\phi_{i}\right)=∥v_{i}-v_{\|}\left(x_{i}\right){∥}^{2}-b\cos\left(\phi_{i},\phi_{i}^{\mathrm{corr}}\right)+\lambda {∥\phi_{i}∥}^{2}$$

Here, $\phi_{i}^{\mathrm{corr}}$ denotes the transition probabilities provided by the Cosine Kernel, and the last term is a regularization term. Thus, $v_{\|}$ serves as a geometry-aware correlation of $v_{i}$, ensuring greater coherence with the underlying manifold structure.

Furthermore, the population dynamics in the feature space can be transferred to the dynamics on the graph. The unbalanced Fokker–Planck Equation (3) could be generalized to a graph, such that the mass evolution at node *i* is given by the following equation:

$$\frac{dp_{i}}{dt}=-\frac{1}{2}\sum_{j\neq i}\left(p_{i}\phi_{ij}-p_{j}\phi_{ij}\right)+\sum_{j\neq i}\frac{D_{ij}}{{\left|e_{ij}\right|}^{2}}\left(p_{j}-p_{i}\right)+g_{i}p_{i}$$

### 5.2. Modeling Cell–Cell Interaction Dynamically

For temporally resolved single-cell RNA-seq data, previous modeling approaches have typically been developed in the continuous space of $R^{d}$. However, the data inherently exist within a discrete space. In addition, incorporating cell–cell communication or interaction into these dynamics are important for constructing accurate spatiotemporal developmental landscapes and for advancing our understanding of complex biological systems [165,166,167,168,169,170,171]. Therefore, an interesting question is how to construct continuous cellular dynamics from a discrete space of interacting cells, e.g., those represented by a graph [172].

In [83], it proposes GraphFP, a graph Fokker–Planck equation-based method to model cellular dynamics by explicitly considering cell interactions. Assume that data can be clustered or annotated into *M* cell types, GraphFP constructs a cell state transition graph G=(V,E), where each vertex in *V* represents a cell type and each edge {i,j} in *E* means the cell type *i* can transit to cell type *j*. Unlike other methods considering probability distribution in $R^{d}$, GraphFP consider the probability distribution on graph *G*. Suppose there are *M* vertices in graph *G*, consider the probability simplex supported on all vertices of *G*

$$P\left(G\right)=\{p\left(t\right)={\left(p_{i}\left(t\right)\right)}_{i=1}^{M}|\sum_{i=1}^{M}p_{i}\left(t\right)=1,p_{i}\left(t\right)\geq 0\}.$$

The aim is also to transport the distribution from $p_{0}$ to $p_{1}$ on *G*, satisfying least action principles. Similar to the continuous space case, one need to define the Fokker–Planck equation and the Wasserstein distance on graph *G*. First, one can define a free energy F:P(G)→R, then the Fokker–Planck equation can be defined as follows:

$$\frac{dp_{i}\left(t\right)}{dt}=\sum_{j\in N(i)}\left(\frac{\partial F(p)}{\partial p_{j}}-\frac{\partial F(p)}{\partial p_{i}}\right)g_{ij}\left(p\right),$$

where N(i) is the neighbor set of vertex *i* and $g_{ij}\left(p\right)$ satisfy certain constraints that could constructed from p [83]. Next, the discrete L2-Wasserstein distance on graph *G* between $p_{0},p_{1}\in P\left(G\right)$ can be defined as

$$W_{2,G}^{2}\left(p_{0},p_{1}\right)=\underset{F}{\inf}\frac{1}{2}\int_{0}^{1}\sum_{i,j\in E}{\left(\frac{\partial F(p)}{\partial p_{j}}-\frac{\partial F(p)}{\partial p_{i}}\right)}^{2}g_{ij}\left(p\right).$$

Next, the target is to find the minimum energy path. In [83], they parameterize F by a linear energy form

$$\begin{array}{cc} F(p|\Phi ,W) & =V(p)+W(p)+\beta H(p),\\ & =\sum_{i=1}^{n}\Phi_{i}p_{i}+\frac{1}{2}\sum_{i=1}^{n}\sum_{j=1}^{n}w_{ij}p_{i}p_{j}+\beta \sum_{i=1}^{n}p_{i}\log p_{i},\\ & =p^{T}\Phi +\frac{1}{2}p^{T}Wp+\beta \sum_{i=1}^{n}p_{i}\log p_{i}, \end{array}$$

where $\Phi ={\left(\Phi_{i}\right)}_{i=1}^{M}$, $W={\left(w_{i,j}\right)}_{1\leq i,j\leq M}$ represents the **interactions** among cell types, and β≥0 is a hyper-parameter. After this parametrization, one denotes the parameters of the free energy as θ={Φ,W}, and the goal is to find the parameter θ such that

$$\begin{array}{cc} & \theta^{*}=\arg\min_{\theta }\;\int_{t_{1}}^{t_{f}}\frac{1}{2}\sum_{\{i,j\}\in E}{\left(\frac{\partial F(p)}{\partial p_{i}}-\frac{\partial F(p)}{\partial p_{j}}\right)}^{2}\cdot g_{ij}\left(p\left(t\right)\right)dt, \end{array}$$

subject to the constraints

$$\begin{array}{cc} \frac{dp(t)}{dt} & ={\left(\sum_{j\in N(i)}\left(\frac{\partial F(p)}{\partial p_{j}}-\frac{\partial F(p)}{\partial p_{i}}\right)g_{ij}\left(p\left(t\right)\right)\right)}_{i=1}^{M}\\ p(1) & =p_{1} \end{array}$$

This problem can be solved by the adjoint method. Once these dynamics are solved, one can then use them for downstream analysis, e.g., cell–cell interaction, probability flow of cell types, and the potential energy [83].

Looking ahead, we anticipate that important future directions based on GraphFP include the expansion of the current framework to achieve single-cell resolution instead of cellular types, incorporating the matching of unnormalized distribution results from cell proliferation and death, and extending the model to spatial transcriptomics.

### 5.3. Reconstructing Waddington Developmental Landscapes

Waddington’s landscape metaphor is a widely recognized framework to depict the cell fate decision process. This conceptual model suggests that metastable cellular states are analogous to wells within a potential landscape, and transitions between these states can be understood as movements or “hops” between these potential wells. While the development of such potential landscapes has been extensively explored [4,64,149,173,174,175,176,177,178,179,180,181,182], effectively constructing these landscapes using single-cell omics data remains the major challenge. In recent works [183,184], the authors utilize RNA velocity to construct a vector field from the snapshot scRNA seq data and then compute the potential landscape based on the Boltzmann distribution-like relations proposed by Wang et al. [185]. To be precise, the landscape is characterized by the expression

$$U=-\sigma^{2}\log p_{\mathrm{ss}}/2$$

where $p_{\mathrm{ss}}$ represents the steady-state probability density function (PDF) that satisfies the steady-state Fokker–Planck equation: $-\nabla \cdot \left(p_{ss}b\right)+\frac{\sigma^{2}}{2}\Delta p_{\mathrm{ss}}=gp_{\mathrm{ss}}$. For the temporal scRNA-seq data, following [40,136], it enables a natural inference of the time-evolving potential energy landscape by leveraging the learned log-density function. Specifically, one can define the landscape at time *t* as

$$U\left(x,t\right)=-\frac{\sigma^{2}\left(t\right)}{2}\log p\left(x,t\right).$$

Regions of lower energy correspond to more stable cell fates, providing a quantitative measure of stability in the cellular state space.

### 5.4. Challenges and Further Directions

Integrating multiomics data is critical for comprehensively characterizing cell–cell interaction dynamics and regulatory mechanisms [186,187,188,189,190,191,192,193,194,195]. Furthermore, aligning both temporal and spatial scales within time-series spatial transcriptomics data (e.g., using non-rigid or non-linear spatial transformations, and latent space) presents a significant challenge. Additionally, the application of SDEs in modeling spatial transcriptomics data, and subsequently constructing spatiotemporal developmental landscapes, represents an important avenue for further exploration.

## 6. Discussion and Conclusions

Inferring dynamical processes from high-throughput single-cell sequencing data is a critical problem in understanding cellular development and fate decisions. With the advancements in sequencing technologies, the field has evolved from dynamic inference based on snapshot single-cell RNA sequencing (scRNA-seq) data to inferring dynamics from temporally resolved scRNA-seq data. Moreover, the development of spatial transcriptomics and time-series spatial transcriptomics (ST) data now offers the potential to decode the spatiotemporal developmental trajectories of single cells and construct their spatiotemporal dynamics. In this review, we have focused on dissecting biological data through the lens of dynamical systems models, specifically investigating how various kinds of models can be applied to study cellular development and fate decisions.

When presenting existing approaches, we chose various types of dynamical systems modeling approaches as the main focus, providing a systematic overview of their applications across different contexts. Specifically, we examine the utility and limitations of dynamic modeling techniques in four distinct data scenarios: (1) single time-point scRNA-seq data, (2) multi time-point scRNA-seq data, (3) single time-point spatial transcriptomics data, and (4) multi time-point spatial transcriptomics data, i.e., spatiotemporal single-cell data. For each data type, we explore how different modeling paradigms—ranging from discrete Markov chain models to continuous Ordinary Differential Equations (ODEs), Stochastic Differential Equations (SDEs), and Partial Differential Equations (PDEs)—can be used to study the underlying biological processes embedded in the snapshot and high-dimensional nature of sequencing data.

For static single snapshot scRNA-seq data, where explicit temporal information is unavailable, top-down discrete-state models such as Markov chains have been widely adopted to infer latent cell state transitions and developmental trajectories. We also address how bottom-up mechanism models like RNA velocity methods provide insights into modeling complex state transition dynamics. When explicit temporal resolution is introduced as in time-series scRNA-seq datasets, continuous dynamical models become more suitable. Incorporating the dynamical optimal transport (OT) theoretical framework, the Fokker–Planck PDE-based models allow us to track the evolution of cellular states over time. The integration of spatial transcriptomics adds another dimension to the modeling challenge. In the case of multi-time-point spatial transcriptomics, we review emerging approaches that combine dynamical OT and geometric transformation to simultaneously account for trajectory inference and batch correction across time points.

Beyond summarizing the mathematical aspects of existing models, we also provide a forward-looking perspective on potential future directions. For instance, integrating both discrete and continuous models, as well as cellular interaction effects, could provide new insights to handle more realistic dynamics with increasing biological interpretability. Additionally, the use of multi-modal omics could also enhance the resolution of dynamical models. To sum up, by examining the intersection of dynamical systems theory and single-cell data modeling, this review provides conceptual and methodological insights that may inspire the development of novel algorithms to dissect the spatiotemporal dynamics underlying single-cell sequencing data.

Due to the limited scope of the current review, several important aspects of the dynamical models of scRNA-seq have not been discussed thoroughly here and remain for further exploration. Firstly, lineage tracing plays a critical role in understanding cellular history and developmental trajectories, and integrating such data into trajectory inference could provide deeper insights into cellular fate decisions [116,196,197]. Secondly, incorporating gene regulatory networks (GRNs) [198,199,200,201,202,203,204] into spatiotemporal trajectory inference is an exciting avenue for future research, as it could enhance the understanding of the regulatory mechanisms driving cellular transitions. Lastly, the concepts of dynamic network biomarkers (DNBs) and critical transitions [205,206,207,208] are promising for understanding cellular fate shifts, particularly in disease progression and cellular development.

Overall, this review demonstrates how dynamic modeling approaches can provide insight into the underlying biological processes underlying single-cell transcriptomics, spatial transcriptomics, and their temporal extensions. In the future, these techniques, when combined with machine learning and other computational advancements, will enable more comprehensive models of cellular dynamics, promising new therapeutic strategies and a deeper understanding of development, disease, and tissue regeneration.

## Abbreviations

The following abbreviations are used in this manuscript:

scRNA-seq	single-cell RNA sequencing
SDE	Stochastic Differential Equation
ODE	Ordinary Differential Equation
PDE	Partial Differential Equation
OT	Optimal Transport
ICA	Independent Component Analysis
PCA	Principle Component Analysis
MST	Minimum Spanning Tree
MCE	Markov Chain Entropy
GPCCA	Generalized Perron Cluster Cluster Analysis
EM	Expectation Maximum
VAE	Variational Autoencoder
RKHS	Reproducing Kernel Hilbert Space
CNF	Continuous Normalizing Flow
FM	Flow Matching
CFM	Conditional Flow Matching
SB	Schrödinger Bridge
RUOT	Regularized Unbalanced Optimal Transport
GWOT	Gromov–Wasserstein optimal transport
FGWOT	Fused Gromov–Wasserstein optimal transport

## References

[1] Lei J. (2023). Mathematical modeling of heterogeneous stem cell regeneration: From cell division to Waddington’s epigenetic landscape. arXiv.

[2] Hong T., Xing J. (2024). Data-and theory-driven approaches for understanding paths of epithelial–mesenchymal transition. Genesis.

[3] Xing J. (2022). Reconstructing data-driven governing equations for cell phenotypic transitions: Integration of data science and systems biology. Phys. Biol..

[4] Schiebinger G. (2021). Reconstructing developmental landscapes and trajectories from single-cell data. Curr. Opin. Syst. Biol..

[5] Heitz M., Ma Y., Kubal S., Schiebinger G. (2024). Spatial Transcriptomics Brings New Challenges and Opportunities for Trajectory Inference. Annu. Rev. Biomed. Data Sci..

[6] Waddington C.H. (2014). The Strategy of the Genes.

[7] Moris N., Pina C., Arias A.M. (2016). Transition states and cell fate decisions in epigenetic landscapes. Nat. Rev. Genet..

[8] MacLean A.L., Hong T., Nie Q. (2018). Exploring intermediate cell states through the lens of single cells. Curr. Opin. Syst. Biol..

[9] Ziegenhain C., Vieth B., Parekh S., Reinius B., Guillaumet-Adkins A., Smets M., Leonhardt H., Heyn H., Hellmann I., Enard W. (2017). Comparative analysis of single-cell RNA sequencing methods. Mol. Cell.

[10] Tang F., Barbacioru C., Wang Y., Nordman E., Lee C., Xu N., Wang X., Bodeau J., Tuch B.B., Siddiqui A. (2009). mRNA-Seq whole-transcriptome analysis of a single cell. Nat. Methods.

[11] Stark R., Grzelak M., Hadfield J. (2019). RNA sequencing: The teenage years. Nat. Rev. Genet..

[12] Ding J., Sharon N., Bar-Joseph Z. (2022). Temporal modelling using single-cell transcriptomics. Nat. Rev. Genet..

[13] Bunne C., Schiebinger G., Krause A., Regev A., Cuturi M. (2024). Optimal transport for single-cell and spatial omics. Nat. Rev. Methods Prim..

[14] Ståhl P.L., Salmén F., Vickovic S., Lundmark A., Navarro J.F., Magnusson J., Giacomello S., Asp M., Westholm J.O., Huss M. (2016). Visualization and analysis of gene expression in tissue sections by spatial transcriptomics. Science.

[15] Rodriques S.G., Stickels R.R., Goeva A., Martin C.A., Murray E., Vanderburg C.R., Welch J., Chen L.M., Chen F., Macosko E.Z. (2019). Slide-seq: A scalable technology for measuring genome-wide expression at high spatial resolution. Science.

[16] Stickels R.R., Murray E., Kumar P., Li J., Marshall J.L., Di Bella D.J., Arlotta P., Macosko E.Z., Chen F. (2021). Highly sensitive spatial transcriptomics at near-cellular resolution with Slide-seqV2. Nat. Biotechnol..

[17] Chen A., Liao S., Cheng M., Ma K., Wu L., Lai Y., Qiu X., Yang J., Xu J., Hao S. (2022). Spatiotemporal transcriptomic atlas of mouse organogenesis using DNA nanoball-patterned arrays. Cell.

[18] Oliveira M.F., Romero J.P., Chung M., Williams S., Gottscho A.D., Gupta A., Pilipauskas S.E., Mohabbat S., Raman N., Sukovich D. (2024). Characterization of immune cell populations in the tumor microenvironment of colorectal cancer using high definition spatial profiling. bioRxiv.

[19] Moffitt J.R., Bambah-Mukku D., Eichhorn S.W., Vaughn E., Shekhar K., Perez J.D., Rubinstein N.D., Hao J., Regev A., Dulac C. (2018). Molecular, spatial, and functional single-cell profiling of the hypothalamic preoptic region. Science.

[20] Eng C.H.L., Lawson M., Zhu Q., Dries R., Koulena N., Takei Y., Yun J., Cronin C., Karp C., Yuan G.C. (2019). Transcriptome-scale super-resolved imaging in tissues by RNA seqFISH+. Nature.

[21] Wang X., Allen W.E., Wright M.A., Sylwestrak E.L., Samusik N., Vesuna S., Evans K., Liu C., Ramakrishnan C., Liu J. (2018). Three-dimensional intact-tissue sequencing of single-cell transcriptional states. Science.

[22] Liu L., Chen A., Li Y., Mulder J., Heyn H., Xu X. (2024). Spatiotemporal omics for biology and medicine. Cell.

[23] Qiu X., Mao Q., Tang Y., Wang L., Chawla R., Pliner H.A., Trapnell C. (2017). Reversed graph embedding resolves complex single-cell trajectories. Nat. Methods.

[24] Cao J., Spielmann M., Qiu X., Huang X., Ibrahim D.M., Hill A.J., Zhang F., Mundlos S., Christiansen L., Steemers F.J. (2019). The single-cell transcriptional landscape of mammalian organogenesis. Nature.

[25] Street K., Risso D., Fletcher R.B., Das D., Ngai J., Yosef N., Purdom E., Dudoit S. (2018). Slingshot: Cell lineage and pseudotime inference for single-cell transcriptomics. BMC Genom..

[26] La Manno G., Soldatov R., Zeisel A., Braun E., Hochgerner H., Petukhov V., Lidschreiber K., Kastriti M.E., Lönnerberg P., Furlan A. (2018). RNA velocity of single cells. Nature.

[27] Bergen V., Soldatov R.A., Kharchenko P.V., Theis F.J. (2021). RNA velocity—current challenges and future perspectives. Mol. Syst. Biol..

[28] Wang K., Hou L., Wang X., Zhai X., Lu Z., Zi Z., Zhai W., He X., Curtis C., Zhou D. (2024). PhyloVelo enhances transcriptomic velocity field mapping using monotonically expressed genes. Nat. Biotechnol..

[29] Liu Y., Huang K., Chen W. (2024). Resolving cellular dynamics using single-cell temporal transcriptomics. Curr. Opin. Biotechnol..

[30] Bergen V., Lange M., Peidli S., Wolf F.A., Theis F.J. (2020). Generalizing RNA velocity to transient cell states through dynamical modeling. Nat. Biotechnol..

[31] Ho J., Jain A., Abbeel P. (2020). Denoising diffusion probabilistic models. Advances in Neural Information Processing Systems.

[32] Sohl-Dickstein J., Weiss E., Maheswaranathan N., Ganguli S. Deep unsupervised learning using nonequilibrium thermodynamics. Proceedings of the International Conference on Machine Learning, PMLR.

[33] Song Y., Sohl-Dickstein J., Kingma D.P., Kumar A., Ermon S., Poole B. Score-Based Generative Modeling through Stochastic Differential Equations. Proceedings of the International Conference on Learning Representations.

[34] Ren T., Zhang Z., Li Z., Jiang J., Qin S., Li G., Li Y., Zheng Y., Li X., Zhan M. (2025). Zeroth-order Informed Fine-Tuning for Diffusion Model: A Recursive Likelihood Ratio Optimizer. arXiv.

[35] Schiebinger G., Shu J., Tabaka M., Cleary B., Subramanian V., Solomon A., Gould J., Liu S., Lin S., Berube P. (2019). Optimal-transport analysis of single-cell gene expression identifies developmental trajectories in reprogramming. Cell.

[36] Klein D., Palla G., Lange M., Klein M., Piran Z., Gander M., Meng-Papaxanthos L., Sterr M., Saber L., Jing C. (2025). Mapping cells through time and space with moscot. Nature.

[37] Lipman Y., Chen R.T.Q., Ben-Hamu H., Nickel M., Le M. Flow Matching for Generative Modeling. Proceedings of the Eleventh International Conference on Learning Representations.

[38] Tong A., FATRAS K., Malkin N., Huguet G., Zhang Y., Rector-Brooks J., Wolf G., Bengio Y. (2024). Improving and generalizing flow-based generative models with minibatch optimal transport. Trans. Mach. Learn. Res..

[39] Gentil I., Léonard C., Ripani L. (2017). About the analogy between optimal transport and minimal entropy. Ann. Fac. Sci. Toulouse Math..

[40] Zhang Z., Li T., Zhou P. Learning stochastic dynamics from snapshots through regularized unbalanced optimal transport. Proceedings of the Thirteenth International Conference on Learning Representations.

[41] Peng Q., Zhou P., Li T. (2024). stVCR: Reconstructing spatio-temporal dynamics of cell development using optimal transport. bioRxiv.

[42] Wang L., Zhang Q., Qin Q., Trasanidis N., Vinyard M., Chen H., Pinello L. (2021). Current progress and potential opportunities to infer single-cell developmental trajectory and cell fate. Curr. Opin. Syst. Biol..

[43] Saelens W., Cannoodt R., Todorov H., Saeys Y. (2019). A comparison of single-cell trajectory inference methods. Nat. Biotechnol..

[44] Li T., Shi J., Wu Y., Zhou P. (2020). On the mathematics of RNA velocity I: Theoretical analysis. bioRxiv.

[45] Jiang Q., Wan L. (2025). Dynamic modeling, optimization, and deep learning for high-dimensional complex biological data. Sci. Sin. Math..

[46] Deconinck L., Cannoodt R., Saelens W., Deplancke B., Saeys Y. (2021). Recent advances in trajectory inference from single-cell omics data. Curr. Opin. Syst. Biol..

[47] Gandrillon O., Gaillard M., Espinasse T., Garnier N.B., Dussiau C., Kosmider O., Sujobert P. (2021). Entropy as a measure of variability and stemness in single-cell transcriptomics. Curr. Opin. Syst. Biol..

[48] Grün D., Muraro M.J., Boisset J.C., Wiebrands K., Lyubimova A., Dharmadhikari G., van den Born M., Van Es J., Jansen E., Clevers H. (2016). De novo prediction of stem cell identity using single-cell transcriptome data. Cell Stem Cell.

[49] Guo M., Bao E.L., Wagner M., Whitsett J.A., Xu Y. (2017). SLICE: Determining cell differentiation and lineage based on single cell entropy. Nucleic Acids Res..

[50] Teschendorff A.E., Enver T. (2017). Single-cell entropy for accurate estimation of differentiation potency from a cell’s transcriptome. Nat. Commun..

[51] Shi J., Teschendorff A.E., Chen W., Chen L., Li T. (2020). Quantifying Waddington’s epigenetic landscape: A comparison of single-cell potency measures. Briefings Bioinform..

[52] Jin S., MacLean A.L., Peng T., Nie Q. (2018). scEpath: Energy landscape-based inference of transition probabilities and cellular trajectories from single-cell transcriptomic data. Bioinformatics.

[53] Liu J., Song Y., Lei J. (2020). Single-cell entropy to quantify the cellular order parameter from single-cell RNA-seq data. Biophys. Rev. Lett..

[54] Haghverdi L., Büttner M., Wolf F.A., Buettner F., Theis F.J. (2016). Diffusion pseudotime robustly reconstructs lineage branching. Nat. Methods.

[55] Wolf F.A., Hamey F.K., Plass M., Solana J., Dahlin J.S., Göttgens B., Rajewsky N., Simon L., Theis F.J. (2019). PAGA: Graph abstraction reconciles clustering with trajectory inference through a topology preserving map of single cells. Genome Biol..

[56] Coifman R.R., Lafon S. (2006). Diffusion maps. Appl. Comput. Harmon. Anal..

[57] Setty M., Kiseliovas V., Levine J., Gayoso A., Mazutis L., Pe’Er D. (2019). Characterization of cell fate probabilities in single-cell data with Palantir. Nat. Biotechnol..

[58] Stassen S.V., Yip G.G., Wong K.K., Ho J.W., Tsia K.K. (2021). Generalized and scalable trajectory inference in single-cell omics data with VIA. Nat. Commun..

[59] Pandey K., Zafar H. (2022). Inference of cell state transitions and cell fate plasticity from single-cell with MARGARET. Nucleic Acids Res..

[60] Weinreb C., Wolock S., Tusi B.K., Socolovsky M., Klein A.M. (2018). Fundamental limits on dynamic inference from single-cell snapshots. Proc. Natl. Acad. Sci. USA.

[61] Zhou P., Wang S., Li T., Nie Q. (2021). Dissecting transition cells from single-cell transcriptome data through multiscale stochastic dynamics. Nat. Commun..

[62] Lange M., Bergen V., Klein M., Setty M., Reuter B., Bakhti M., Lickert H., Ansari M., Schniering J., Schiller H.B. (2022). CellRank for directed single-cell fate mapping. Nat. Methods.

[63] Weiler P., Lange M., Klein M., Pe’er D., Theis F. (2024). CellRank 2: Unified fate mapping in multiview single-cell data. Nat. Methods.

[64] Zhou P., Li T. (2016). Construction of the landscape for multi-stable systems: Potential landscape, quasi-potential, A-type integral and beyond. J. Chem. Phys..

[65] Vanden-Eijnden E. (2010). Transition-path theory and path-finding algorithms for the study of rare events. Annu. Rev. Phys. Chem..

[66] Reuter B., Fackeldey K., Weber M. (2019). Generalized Markov modeling of nonreversible molecular kinetics. J. Chem. Phys..

[67] Gao M., Qiao C., Huang Y. (2022). UniTVelo: Temporally unified RNA velocity reinforces single-cell trajectory inference. Nat. Commun..

[68] Li J., Pan X., Yuan Y., Shen H.B. (2024). TFvelo: Gene regulation inspired RNA velocity estimation. Nat. Commun..

[69] Oller-Moreno S., Kloiber K., Machart P., Bonn S. (2021). Algorithmic advances in machine learning for single-cell expression analysis. Curr. Opin. Syst. Biol..

[70] Raimundo F., Meng-Papaxanthos L., Vallot C., Vert J.P. (2021). Machine learning for single-cell genomics data analysis. Curr. Opin. Syst. Biol..

[71] Gu Y., Blaauw D., Welch J.D. (2022). Bayesian inference of rna velocity from multi-lineage single-cell data. bioRxiv.

[72] Kingma D.P., Welling M. (2013). Auto-encoding variational bayes. arXiv.

[73] Qiao C., Huang Y. (2021). Representation learning of RNA velocity reveals robust cell transitions. Proc. Natl. Acad. Sci. USA.

[74] Farrell S., Mani M., Goyal S. (2023). Inferring single-cell transcriptomic dynamics with structured latent gene expression dynamics. Cell Rep. Methods.

[75] Gayoso A., Weiler P., Lotfollahi M., Klein D., Hong J., Streets A., Theis F.J., Yosef N. (2024). Deep generative modeling of transcriptional dynamics for RNA velocity analysis in single cells. Nat. Methods.

[76] Gu Y., Blaauw D.T., Welch J. Variational mixtures of ODEs for inferring cellular gene expression dynamics. Proceedings of the International Conference on Machine Learning. PMLR.

[77] Cui H., Maan H., Vladoiu M.C., Zhang J., Taylor M.D., Wang B. (2024). DeepVelo: Deep learning extends RNA velocity to multi-lineage systems with cell-specific kinetics. Genome Biol..

[78] Li S., Zhang P., Chen W., Ye L., Brannan K.W., Le N.T., Abe J.i., Cooke J.P., Wang G. (2024). A relay velocity model infers cell-dependent RNA velocity. Nat. Biotechnol..

[79] Qiu X., Zhang Y., Martin-Rufino J.D., Weng C., Hosseinzadeh S., Yang D., Pogson A.N., Hein M.Y., Min K.H.J., Wang L. (2022). Mapping transcriptomic vector fields of single cells. Cell.

[80] Chen Z., King W.C., Hwang A., Gerstein M., Zhang J. (2022). DeepVelo: Single-cell transcriptomic deep velocity field learning with neural ordinary differential equations. Sci. Adv..

[81] Sha Y., Qiu Y., Zhou P., Nie Q. (2024). Reconstructing growth and dynamic trajectories from single-cell transcriptomics data. Nat. Mach. Intell..

[82] Lavenant H., Zhang S., Kim Y.H., Schiebinger G. (2024). Toward a mathematical theory of trajectory inference. Ann. Appl. Probab..

[83] Jiang Q., Zhang S., Wan L. (2022). Dynamic inference of cell developmental complex energy landscape from time series single-cell transcriptomic data. PLoS Comput. Biol..

[84] Jiang Q., Wan L. (2024). A physics-informed neural SDE network for learning cellular dynamics from time-series scRNA-seq data. Bioinformatics.

[85] Bunne C., Stark S.G., Gut G., Del Castillo J.S., Levesque M., Lehmann K.V., Pelkmans L., Krause A., Rätsch G. (2023). Learning single-cell perturbation responses using neural optimal transport. Nat. Methods.

[86] Tong A., Kuchroo M., Gupta S., Venkat A., San Juan B.P., Rangel L., Zhu B., Lock J.G., Chaffer C.L., Krishnaswamy S. (2023). Learning transcriptional and regulatory dynamics driving cancer cell plasticity using neural ODE-based optimal transport. bioRxiv.

[87] Zhang S., Afanassiev A., Greenstreet L., Matsumoto T., Schiebinger G. (2021). Optimal transport analysis reveals trajectories in steady-state systems. PLoS Comput. Biol..

[88] Maddu S., Chardès V., Shelley M. (2024). Inferring biological processes with intrinsic noise from cross-sectional data. arXiv.

[89] Eyring L., Klein D., Uscidda T., Palla G., Kilbertus N., Akata Z., Theis F.J. Unbalancedness in Neural Monge Maps Improves Unpaired Domain Translation. Proceedings of the Twelfth International Conference on Learning Representations.

[90] Zhang J., Larschan E., Bigness J., Singh R. (2024). scNODE: Generative model for temporal single cell transcriptomic data prediction. Bioinformatics.

[91] Yeo G.H.T., Saksena S.D., Gifford D.K. (2021). Generative modeling of single-cell time series with PRESCIENT enables prediction of cell trajectories with interventions. Nat. Commun..

[92] Flamary R., Courty N., Gramfort A., Alaya M.Z., Boisbunon A., Chambon S., Chapel L., Corenflos A., Fatras K., Fournier N. (2021). POT: Python Optimal Transport. J. Mach. Learn. Res..

[93] Peyré G., Cuturi M. (2019). Computational optimal transport: With applications to data science. Found. Trends® Mach. Learn..

[94] Benamou J.D., Brenier Y. (2000). A computational fluid mechanics solution to the Monge-Kantorovich mass transfer problem. Numer. Math..

[95] Tong A., Huang J., Wolf G., Van Dijk D., Krishnaswamy S. Trajectorynet: A dynamic optimal transport network for modeling cellular dynamics. Proceedings of the International Conference on Machine Learning. PMLR.

[96] Huguet G., Magruder D.S., Tong A., Fasina O., Kuchroo M., Wolf G., Krishnaswamy S. (2022). Manifold interpolating optimal-transport flows for trajectory inference. Advances in Neural Information Processing Systems.

[97] Ruthotto L., Osher S.J., Li W., Nurbekyan L., Fung S.W. (2020). A machine learning framework for solving high-dimensional mean field game and mean field control problems. Proc. Natl. Acad. Sci. USA.

[98] Liu S., Ma S., Chen Y., Zha H., Zhou H. (2021). Learning high dimensional Wasserstein geodesics. arXiv.

[99] Cheng Q., Liu Q., Chen W., Shen J. (2024). A new flow dynamic approach for Wasserstein gradient flows. arXiv.

[100] Wan W., Zhang Y., Bao C., Dong B., Shi Z. (2023). A scalable deep learning approach for solving high-dimensional dynamic optimal transport. SIAM J. Sci. Comput..

[101] Pooladian A.A., Domingo-Enrich C., Chen R.T.Q., Amos B. Neural Optimal Transport with Lagrangian Costs. Proceedings of the 40th Conference on Uncertainty in Artificial Intelligence.

[102] Klein D., Uscidda T., Theis F., Cuturi M. (2023). Generative Entropic Neural Optimal Transport To Map Within and Across Spaces. arXiv.

[103] Albergo M.S., Vanden-Eijnden E. Building Normalizing Flows with Stochastic Interpolants. Proceedings of the Eleventh International Conference on Learning Representations.

[104] Liu X., Gong C., Liu Q. Flow Straight and Fast: Learning to Generate and Transfer Data with Rectified Flow. Proceedings of the Eleventh International Conference on Learning Representations.

[105] Jiao Y., Lai Y., Wang Y., Yan B. (2024). Convergence Analysis of Flow Matching in Latent Space with Transformers. arXiv.

[106] Gao Y., Huang J., Jiao Y., Zheng S. (2024). Convergence of Continuous Normalizing Flows for Learning Probability Distributions. arXiv.

[107] Liu S., Li W., Zha H., Zhou H. (2022). Neural Parametric Fokker–Planck Equation. SIAM J. Numer. Anal..

[108] Wu H., Liu S., Ye X., Zhou H. (2023). Parameterized wasserstein hamiltonian flow. arXiv.

[109] Jin Y., Liu S., Wu H., Ye X., Zhou H. (2024). Parameterized Wasserstein Gradient Flow. arXiv.

[110] Chow S.N., Li W., Zhou H. (2020). Wasserstein hamiltonian flows. J. Differ. Equ..

[111] Cheng X., Lu J., Tan Y., Xie Y. (2024). Convergence of flow-based generative models via proximal gradient descent in Wasserstein space. IEEE Trans. Inf. Theory.

[112] Zhou P., Gao X., Li X., Li L., Niu C., Ouyang Q., Lou H., Li T., Li F. (2021). Stochasticity triggers activation of the S-phase checkpoint pathway in budding yeast. Phys. Rev. X.

[113] Elowitz M.B., Levine A.J., Siggia E.D., Swain P.S. (2002). Stochastic gene expression in a single cell. Science.

[114] Léonard C. (2014). A survey of the Schrödinger problem and some of its connections with optimal transport. Discret. Contin. Dyn. Syst.-Ser. A.

[115] Pariset M., Hsieh Y.P., Bunne C., Krause A., Bortoli V.D. Unbalanced Diffusion Schrödinger Bridge. Proceedings of the ICML Workshop on New Frontiers in Learning, Control, and Dynamical Systems.

[116] Ventre E., Forrow A., Gadhiwala N., Chakraborty P., Angel O., Schiebinger G. (2023). Trajectory inference for a branching SDE model of cell differentiation. arXiv.

[117] Chizat L., Zhang S., Heitz M., Schiebinger G. (2022). Trajectory inference via mean-field langevin in path space. Advances in Neural Information Processing Systems.

[118] Shi Y., De Bortoli V., Campbell A., Doucet A. (2024). Diffusion Schrödinger bridge matching. Advances in Neural Information Processing Systems.

[119] De Bortoli V., Thornton J., Heng J., Doucet A. (2021). Diffusion schrödinger bridge with applications to score-based generative modeling. Advances in Neural Information Processing Systems.

[120] Pooladian A.A., Niles-Weed J. (2024). Plug-in estimation of Schrödinger bridges. arXiv.

[121] Liu G.H., Chen T., So O., Theodorou E. (2022). Deep Generalized Schrödinger Bridge. Advances in Neural Information Processing Systems.

[122] Gu A., Chien E., Greenewald K. (2024). Partially Observed Trajectory Inference using Optimal Transport and a Dynamics Prior. arXiv.

[123] Koshizuka T., Sato I. Neural Lagrangian Schrödinger Bridge: Diffusion Modeling for Population Dynamics. Proceedings of the Eleventh International Conference on Learning Representations.

[124] Neklyudov K., Brekelmans R., Severo D., Makhzani A. Action matching: Learning stochastic dynamics from samples. Proceedings of the International Conference on Machine Learning. PMLR.

[125] Neklyudov K., Brekelmans R., Tong A., Atanackovic L., Liu Q., Makhzani A. A Computational Framework for Solving Wasserstein Lagrangian Flows. Proceedings of the Forty-first International Conference on Machine Learning.

[126] Zhang P., Gao T., Guo J., Duan J. (2024). Action Functional as Early Warning Indicator in the Space of Probability Measures. arXiv.

[127] Bunne C., Hsieh Y.P., Cuturi M., Krause A. The schrödinger bridge between gaussian measures has a closed form. Proceedings of the International Conference on Artificial Intelligence and Statistics, PMLR.

[128] Chen T., Liu G.H., Theodorou E. Likelihood Training of Schrödinger Bridge using Forward-Backward SDEs Theory. Proceedings of the International Conference on Learning Representations.

[129] Albergo M.S., Boffi N.M., Vanden-Eijnden E. (2023). Stochastic interpolants: A unifying framework for flows and diffusions. arXiv.

[130] Wang G., Jiao Y., Xu Q., Wang Y., Yang C. Deep generative learning via schrödinger bridge. Proceedings of the International Conference on Machine Learning, PMLR.

[131] Jiao Y., Kang L., Lin H., Liu J., Zuo H. (2024). Latent Schrödinger Bridge Diffusion Model for Generative Learning. arXiv.

[132] Zhou L., Lou A., Khanna S., Ermon S. Denoising Diffusion Bridge Models. Proceedings of the Twelfth International Conference on Learning Representations.

[133] Liu G.H., Vahdat A., Huang D.A., Theodorou E.A., Nie W., Anandkumar A. (2023). I^2^SB: Image-to-Image Schrödinger Bridge. arXiv.

[134] Zhou M., Osher S., Li W. (2024). Score-based Neural Ordinary Differential Equations for Computing Mean Field Control Problems. arXiv.

[135] Zhu Q., Zhao B., Zhang J., Li P., Lin W. (2024). Governing equation discovery of a complex system from snapshots. arXiv.

[136] Tong A., Malkin N., Fatras K., Atanackovic L., Zhang Y., Huguet G., Wolf G., Bengio Y. Simulation-Free Schrödinger Bridges via Score and Flow Matching. Proceedings of the International Conference on Artificial Intelligence and Statistics, PMLR.

[137] Chen Y., Georgiou T.T., Pavon M. (2022). The most likely evolution of diffusing and vanishing particles: Schrodinger bridges with unbalanced marginals. SIAM J. Control Optim..

[138] Baradat A., Lavenant H. (2021). Regularized unbalanced optimal transport as entropy minimization with respect to branching brownian motion. arXiv.

[139] Buze M., Duong M.H. (2023). Entropic regularisation of unbalanced optimal transportation problems. arXiv.

[140] Janati H., Muzellec B., Peyré G., Cuturi M. (2020). Entropic optimal transport between unbalanced gaussian measures has a closed form. Advances in Neural Information Processing Systems.

[141] Chen R.T.Q., Rubanova Y., Bettencourt J., Duvenaud D. (2018). Neural Ordinary Differential Equations. Advances in Neural Information Processing Systems.

[142] Dai Pra P. (1991). A stochastic control approach to reciprocal diffusion processes. Appl. Math. Optim..

[143] Chen Y., Georgiou T.T., Pavon M. (2016). On the relation between optimal transport and Schrödinger bridges: A stochastic control viewpoint. J. Optim. Theory Appl..

[144] Chizat L., Peyré G., Schmitzer B., Vialard F.X. (2018). An interpolating distance between optimal transport and Fisher–Rao metrics. Found. Comput. Math..

[145] Chizat L., Peyré G., Schmitzer B., Vialard F.X. (2018). Unbalanced optimal transport: Dynamic and Kantorovich formulations. J. Funct. Anal..

[146] Gangbo W., Li W., Osher S., Puthawala M. (2019). Unnormalized optimal transport. J. Comput. Phys..

[147] Raissi M., Perdikaris P., Karniadakis G.E. (2019). Physics-informed neural networks: A deep learning framework for solving forward and inverse problems involving nonlinear partial differential equations. J. Comput. Phys..

[148] Pham D., Tan X., Balderson B., Xu J., Grice L.F., Yoon S., Willis E.F., Tran M., Lam P.Y., Raghubar A. (2023). Robust mapping of spatiotemporal trajectories and cell–cell interactions in healthy and diseased tissues. Nat. Commun..

[149] Zhou P., Bocci F., Li T., Nie Q. (2024). Spatial transition tensor of single cells. Nat. Methods.

[150] Shen X., Zuo L., Ye Z., Yuan Z., Huang K., Li Z., Yu Q., Zou X., Wei X., Xu P. (2025). Inferring cell trajectories of spatial transcriptomics via optimal transport analysis. Cell Syst..

[151] Tan Y., Wang A., Wang Z., Lin W., Yan Y., Nie Q., Shi J. (2024). Transfer learning of multicellular organization via single-cell and spatial transcriptomics. bioRxiv.

[152] Gu Y., Liu J., Li C., Welch J.D. (2024). Mapping Cell Fate Transition in Space and Time. Proceedings of the International Conference on Research in Computational Molecular Biology.

[153] Qiu X., Zhu D.Y., Yao J., Jing Z., Zuo L., Wang M., Min K.H., Pan H., Wang S., Liao S. (2022). Spateo: Multidimensional spatiotemporal modeling of single-cell spatial transcriptomics. bioRxiv.

[154] Wei X., Fu S., Li H., Liu Y., Wang S., Feng W., Yang Y., Liu X., Zeng Y.Y., Cheng M. (2022). Single-cell Stereo-seq reveals induced progenitor cells involved in axolotl brain regeneration. Science.

[155] Wang M., Hu Q., Tu Z., Kong L., Yao J., Xiang R., Chen Z., Zhao Y., Zhou Y., Yu T. (2024). A single-cell 3D spatiotemporal multi-omics atlas from Drosophila embryogenesis to metamorphosis. bioRxiv.

[156] Zeira R., Land M., Strzalkowski A., Raphael B.J. (2022). Alignment and integration of spatial transcriptomics data. Nat. Methods.

[157] Halmos P., Liu X., Gold J., Chen F., Ding L., Raphael B.J. (2024). DeST-OT: Alignment of Spatiotemporal Transcriptomics Data. bioRxiv.

[158] Titouan V., Courty N., Tavenard R., Flamary R. Optimal transport for structured data with application on graphs. Proceedings of the International Conference on Machine Learning, PMLR.

[159] Chowdhury S., Mémoli F. (2019). The Gromov–Wasserstein distance between networks and stable network invariants. Inf. Inference J. IMA.

[160] Cohen S., Guibasm L., Werner B. (1999). The earth mover’s distance under transformation sets. Proceedings of the Seventh IEEE International Conference on Computer Vision.

[161] Coifman R.R., Kevrekidis I.G., Lafon S., Maggioni M., Nadler B. (2008). Diffusion maps, reduction coordinates, and low dimensional representation of stochastic systems. Multiscale Model. Simul..

[162] Hetzel L., Fischer D.S., Günnemann S., Theis F.J. (2021). Graph representation learning for single-cell biology. Curr. Opin. Syst. Biol..

[163] Zhang Y., Qiu X., Ni K., Weissman J., Bahar I., Xing J. (2023). Graph-Dynamo: Learning stochastic cellular state transition dynamics from single cell data. bioRxiv.

[164] Chen Y., Zhang Y., Gan J., Ni K., Chen M., Bahar I., Xing J. (2024). GraphVelo allows inference of multi-modal single cell velocities and molecular mechanisms. bioRxiv.

[165] Almet A.A., Cang Z., Jin S., Nie Q. (2021). The landscape of cell–cell communication through single-cell transcriptomics. Curr. Opin. Syst. Biol..

[166] Jin S., Guerrero-Juarez C.F., Zhang L., Chang I., Ramos R., Kuan C.H., Myung P., Plikus M.V., Nie Q. (2021). Inference and analysis of cell-cell communication using CellChat. Nat. Commun..

[167] Jin S., Plikus M.V., Nie Q. (2025). CellChat for systematic analysis of cell–cell communication from single-cell transcriptomics. Nat. Protoc..

[168] Cang Z., Zhao Y., Almet A.A., Stabell A., Ramos R., Plikus M.V., Atwood S.X., Nie Q. (2023). Screening cell–cell communication in spatial transcriptomics via collective optimal transport. Nat. Methods.

[169] Almet A.A., Tsai Y.C., Watanabe M., Nie Q. (2024). Inferring pattern-driving intercellular flows from single-cell and spatial transcriptomics. Nat. Methods.

[170] Wada T., Hironaka K.i., Kuroda S. (2021). Cell-to-cell variability serves as information not noise. Curr. Opin. Syst. Biol..

[171] Topolewski P., Komorowski M. (2021). Information-theoretic analyses of cellular strategies for achieving high signaling capacity—dynamics, cross-wiring, and heterogeneity of cellular states. Curr. Opin. Syst. Biol..

[172] Gandrillon O., Stumpf M.P. (2021). Editorial overview: ‘Theoretical approaches to analyze single-cell data’ (April 2021) within the theme ‘Mathematical modelling’. Curr. Opin. Syst. Biol..

[173] Ao P. (2004). Potential in stochastic differential equations: Novel construction. J. Phys. A Math. Gen..

[174] Shi J., Aihara K., Li T., Chen L. (2022). Energy landscape decomposition for cell differentiation with proliferation effect. Natl. Sci. Rev..

[175] Zhao Y., Zhang W., Li T. (2024). EPR-Net: Constructing a non-equilibrium potential landscape via a variational force projection formulation. Natl. Sci. Rev..

[176] Li C., Wang J. (2013). Quantifying cell fate decisions for differentiation and reprogramming of a human stem cell network: Landscape and biological paths. PLoS Comput. Biol..

[177] Wang J., Li C., Wang E. (2010). Potential and flux landscapes quantify the stability and robustness of budding yeast cell cycle network. Proc. Natl. Acad. Sci. USA.

[178] Li C., Wang J. (2014). Landscape and flux reveal a new global view and physical quantification of mammalian cell cycle. Proc. Natl. Acad. Sci. USA.

[179] Bian S., Zhang Y., Li C. (2023). An improved approach for calculating energy landscape of gene networks from moment equations. Chaos Interdiscip. J. Nonlinear Sci..

[180] Bian S., Zhou R., Lin W., Li C. (2024). Quantifying energy landscape of oscillatory systems: Explosion, pre-solution, and diffusion decomposition. arXiv.

[181] Zhou R., Yu Y., Li C. (2024). Revealing neural dynamical structure of C. elegans with deep learning. Iscience.

[182] Torregrosa G., Garcia-Ojalvo J. (2021). Mechanistic models of cell-fate transitions from single-cell data. Curr. Opin. Syst. Biol..

[183] Zhu L., Wang J. (2024). Quantifying Landscape-Flux via Single-Cell Transcriptomics Uncovers the Underlying Mechanism of Cell Cycle. Adv. Sci..

[184] Zhu L., Yang S., Zhang K., Wang H., Fang X., Wang J. (2024). Uncovering underlying physical principles and driving forces of cell differentiation and reprogramming from single-cell transcriptomics. Proc. Natl. Acad. Sci. USA.

[185] Wang J., Xu L., Wang E. (2008). Potential landscape and flux framework of nonequilibrium networks: Robustness, dissipation, and coherence of biochemical oscillations. Proc. Natl. Acad. Sci. USA.

[186] Stein-O’Brien G.L., Ainslie M.C., Fertig E.J. (2021). Forecasting cellular states: From descriptive to predictive biology via single-cell multiomics. Curr. Opin. Syst. Biol..

[187] Cang Z., Zhao Y. (2024). Synchronized Optimal Transport for Joint Modeling of Dynamics Across Multiple Spaces. arXiv.

[188] Demetci P., Santorella R., Sandstede B., Noble W.S., Singh R. (2022). SCOT: Single-cell multi-omics alignment with optimal transport. J. Comput. Biol..

[189] Zhou X., Dong K., Zhang S. (2023). Integrating spatial transcriptomics data across different conditions, technologies and developmental stages. Nat. Comput. Sci..

[190] Cao K., Gong Q., Hong Y., Wan L. (2022). A unified computational framework for single-cell data integration with optimal transport. Nat. Commun..

[191] Xia C.R., Cao Z.J., Tu X.M., Gao G. (2023). Spatial-linked alignment tool (SLAT) for aligning heterogenous slices. Nat. Commun..

[192] Gao Z., Cao K., Wan L. (2024). Graspot: A graph attention network for spatial transcriptomics data integration with optimal transport. bioRxiv.

[193] Tang Z., Luo S., Zeng H., Huang J., Sui X., Wu M., Wang X. (2024). Search and match across spatial omics samples at single-cell resolution. Nat. Methods.

[194] Lahat D., Adali T., Jutten C. (2015). Multimodal data fusion: An overview of methods, challenges, and prospects. Proc. IEEE.

[195] Liu X., Zeira R., Raphael B.J. (2023). Partial alignment of multislice spatially resolved transcriptomics data. Genome Res..

[196] Wagner D.E., Klein A.M. (2020). Lineage tracing meets single-cell omics: Opportunities and challenges. Nat. Rev. Genet..

[197] Weinreb C., Rodriguez-Fraticelli A., Camargo F.D., Klein A.M. (2020). Lineage tracing on transcriptional landscapes links state to fate during differentiation. Science.

[198] Pratapa A., Jalihal A.P., Law J.N., Bharadwaj A., Murali T. (2020). Benchmarking algorithms for gene regulatory network inference from single-cell transcriptomic data. Nat. Methods.

[199] Van de Sande B., Flerin C., Davie K., De Waegeneer M., Hulselmans G., Aibar S., Seurinck R., Saelens W., Cannoodt R., Rouchon Q. (2020). A scalable SCENIC workflow for single-cell gene regulatory network analysis. Nat. Protoc..

[200] Zhang S.Y. (2024). Joint trajectory and network inference via reference fitting. arXiv.

[201] Stumpf M.P. (2021). Inferring better gene regulation networks from single-cell data. Curr. Opin. Syst. Biol..

[202] Akers K., Murali T. (2021). Gene regulatory network inference in single-cell biology. Curr. Opin. Syst. Biol..

[203] Zhao W., Larschan E., Sandstede B., Singh R. (2024). Optimal transport reveals dynamic gene regulatory networks via gene velocity estimation. bioRxiv.

[204] Yang M. Topological Schrödinger Bridge Matching. Proceedings of the Thirteenth International Conference on Learning Representations.

[205] Liu X., Chang X., Liu R., Yu X., Chen L., Aihara K. (2017). Quantifying critical states of complex diseases using single-sample dynamic network biomarkers. PLoS Comput. Biol..

[206] Zhang X., Xiao K., Wen Y., Wu F., Gao G., Chen L., Zhou C. (2024). Multi-omics with dynamic network biomarker algorithm prefigures organ-specific metastasis of lung adenocarcinoma. Nat. Commun..

[207] Chen Z., Bai X., Ma L., Wang X., Liu X., Liu Y., Chen L., Wan L. (2018). A branch point on differentiation trajectory is the bifurcating event revealed by dynamical network biomarker analysis of single-cell data. IEEE/ACM Trans. Comput. Biol. Bioinform..

[208] Han C., Zhong J., Zhang Q., Hu J., Liu R., Liu H., Mo Z., Chen P., Ling F. (2022). Development of a dynamic network biomarkers method and its application for detecting the tipping point of prior disease development. Comput. Struct. Biotechnol. J..

